# Design and Analysis of a Data Fusion Scheme in Mobile Wireless Sensor Networks Based on Multi-Protocol Mobile Agents

**DOI:** 10.3390/s17112523

**Published:** 2017-11-03

**Authors:** Chunxue Wu, Wenliang Wu, Caihua Wan, Ernst Bekkering, Naixue Xiong

**Affiliations:** 1 School of Optical-Electrical & Computer Engineering, University of Shanghai for Science & Technology, Shanghai 200093, China; wcx@usst.edu.cn (C.W.); wwl@st.usst.edu.cn (W.W.); yyp872@gmail.com (C.W.); 2 Department of Mathematics and Computer Science, Northeastern State University, Tahlequah, OK 74464, USA; bekkerin@nsuok.edu

**Keywords:** multi-protocol, mobile sensor networks, data fusion, mobile agents, color sensors

## Abstract

Sensors are increasingly used in mobile environments with wireless network connections. Multiple sensor types measure distinct aspects of the same event. Their measurements are then combined to produce integrated, reliable results. As the number of sensors in networks increases, low energy requirements and changing network connections complicate event detection and measurement. We present a data fusion scheme for use in mobile wireless sensor networks with high energy efficiency and low network delays, that still produces reliable results. In the first phase, we used a network simulation where mobile agents dynamically select the next hop migration node based on the stability parameter of the link, and perform the data fusion at the migration node. Agents use the fusion results to decide if it should return the fusion results to the processing center or continue to collect more data. In the second phase. The feasibility of data fusion at the node level is confirmed by an experimental design where fused data from color sensors show near-identical results to actual physical temperatures. These results are potentially important for new large-scale sensor network applications.

## 1. Introduction

In the past, sensors were manually installed in fixed positions. Over the years, the use of sensors has expanded with the use of mobile sensors with wireless connections in Wireless Sensor Networks (WSNs). Combining the mobility with wireless technology has created new challenges. Connections are temporary and more fragile, and mobile sensors have limited energy storage. Mobile Agents (MAs) can alleviate these challenges. A Mobile Agent is a computer program with autonomous mobility function [1], which can gather data while moving in Wireless Sensor Networks [2,3,4,5,6,7,8]. This special software saves its own state, transfers its state to a new host, and resumes its task at the new host. It carries its own routing information, interacts with the environment, and completes its task autonomously [9]. The path can be static using predefined information, or dynamic based on autonomous routing decisions. Agents can process data independently. Mobile agents allow for effective use of local resources, reduced network load, improved network scalability, and increased reliability and real-time performance [10].

Eventually, central network points collect the data. If sensor nodes send data directly to a sink node, the sink can become a bottleneck. Data fusion at the sink can integrate multi-source data to increase its accuracy, completeness, and dependability. Data fusion by mobile agents can decrease energy consumption, increase efficiency, improve precision, and help meet application layer requirements [11]. Movement can interfere with this process when transmission of routing information [12] and data [13] is interrupted. In that case, data fusion in Mobile Wireless Sensor Networks (MWSNs) will suffer from network delays, high and uneven energy consumption, poor scalability, and increased demands on the sensors.

Obviously, sensors must use their limited energy independently and effectively. However, just focusing on energy conservation can result in an ineffective network structure if the conservation efforts do not realize the benefits. Collaborative data mining and information fusion may require sensor interaction to complete tasks or respond to queries. If too many sensors are involved, merging of mined data leads to increased energy consumption and competition for communication resources. On the other hand, decentralization to limit energy consumption and communication may be inaccurate and ineffective due to lack of coordination. Balancing communication requirements and energy consumption with coordination is necessary to maximize use of network information. Our data fusion algorithm for multi-protocol mobile agents in wireless networks contributes to the solution of this dilemma. It is based on the Agent-based Model in cooperative industrial networks [14], which shows the impact of business interoperability on the performance of networked companies. This theoretical agent-based model was confirmed in a dam construction project, where a cooperative information system platform with Radio Frequency Identification (RFI) technology reduced the time for analysis of testing results for concrete up to 98%. Like the earlier study, we developed a simulation model and confirmed the model experimentally. Sending data by mobile agents in fused format produces reliable data while lowering energy consumption and network delays, especially in larger networks. These results are potentially important in emerging large-scale mobile wireless networks.

We organize the rest of this paper as follows: Section 2 discusses the related literature and briefly reviews the theoretical framework. Section 3 presents our Improved Multi Mobile Agent Data Fusion scheme. This is a network simulation comparing energy requirements and network delays between fused and unfused data, and with our improved routing decision making. Section 4 discusses the experimental environment where we check if fused data can give reliable measurements of events. Finally, Section 5 reviews our conclusions and Section 6 considers future research opportunities.

## 2. Literature Review

### 2.1. Data Fusion in Sensor Networks

The number of sensors in network grows, sensors may come from multiple sources, and sensors may collect distinct aspects of the same phenomenon. Data needs to be integrated to produce consistent, correct, and more useful information.

#### 2.1.1. Definition and Function of Data Fusion

In 1985, the Joint Directors of Laboratories’ Data Fusion Subpanel (later known as the Data Fusion Group) initially proposed the data fusion model. The current model has six levels, ranging from Source Preprocessing/subject Assessment (level 0) to User/Cognitive Refinement (level 5). The model describes the process of correlating and synthesizing data and information from single or multiple sources to complete correct location and identity estimates with a complete and prompt assessment of data and physical states. The process uses continuous estimation and evaluation optimization, and the process adjusts itself to achieve better results.

Wireless sensor networks are more focused on task completion than care for individual nodes. The network is like a server receiving a request, parsing the query language, informing the network, and returning the results. The focus on purely data-oriented communication is a basic feature of wireless sensor networks. Data processing and fusion from multiple sources is an indispensable part of the network, and brings it in line with the data needed by the user. After fusion, the network can compress data to reduce the amount of information. The main goal of data fusion in sensor networks is reduction of energy consumption, enhanced accuracy, and improved efficiency [15].

#### 2.1.2. Data Fusion Architecture

**Figure 1:**
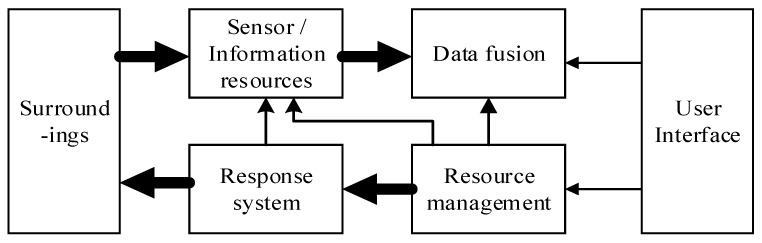
Typical application of information fusion.

Like other information systems, data fusion systems are modular. We show the typical role of data fusion in Figure 1. The system, in this case a combat system, gives the user an assessment of the observed environment. The user further evaluates and controls the response to the assessment. The resource management system uses the merged decision and user instructions to plan and control the available resources. These include combat platform navigation, countermeasures and other active responses, sensors and processing resources for mission tasks, and processing resources for data fusion and response management.

The open system environment is a reference model for systems architecture. The model includes interfaces, tasks, supporting structures, and users. An open system environment should have distributed processing, open hierarchies, recent real-time data distribution, data source independence, software modularity, application independence and scalability of system scale. Its purpose is to reduce development costs, integration costs, and software maintenance costs [16].

#### 2.1.3. Typical Routing Protocols

The network collects sensor information, but mobile wireless networks do not have a fixed topology. We review typical routing protocols to send requests to sensors for data collection and receive data back from the sensors.

##### Query-Based Routing

**Figure 2:**
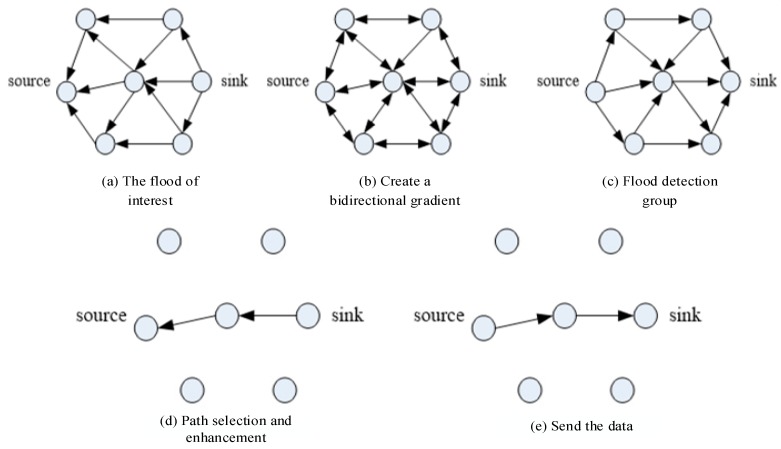
Directional diffusion process.

In query-based routing, the base station broadcasts the interest message to all sensing nodes to set up the path of data transmission and fusion. The interest message includes an inquiry request with list of attributes, duration of the request, and geographical area. Intermediate nodes on the path to the monitoring area cache the interest message and calculate the data gradient, including the data reporting rate and the next hop node. They use the path establishment mechanism to plan the optimized path to the base station, but also several sub-optimal paths to the base station. The base station may send a message of increased or decreased interest in the data sent by a path to change the data reporting rate. Data fusion of the directional diffusion route adopts the method of “suppressing the replica”, that is, nodes buffer only new retrieved data, and do not use duplicate data. This approach is not only simple, but the routing technology can effectively reduce the amount of data in the network [17]. Having multiple paths ensures the robustness of the process, but it is not suitable for large-scale sensor networks due to the cost of building the data gradient. Figure 2 shows the stages of setting up the transmission path.

##### Hierarchical Routing

**Figure 3:**
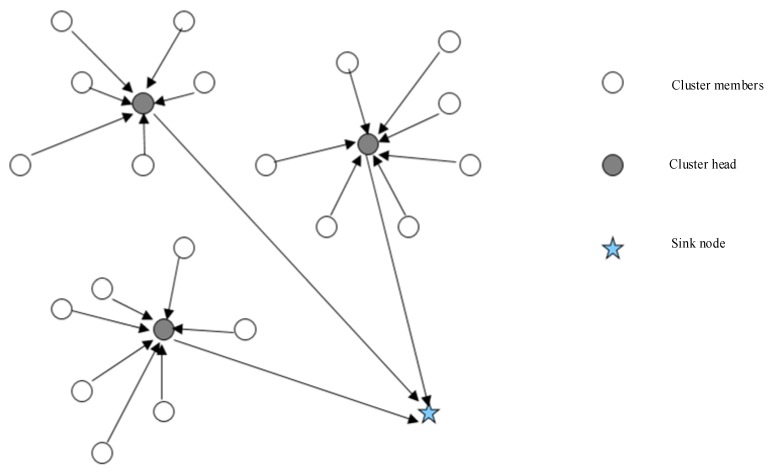
Data fusion in hierarchical routing.

Hierarchical routing is the method of network routing based on hierarchical addressing. Backbones connect routers, which in turn service a group. Both the Low Energy Adaptive Clustering Hierarchy (LEACH) [18] and Low Energy Adaptive Clustering Hierarchy-centralized (LEACH-C) use hierarchical routing. (Figure 3). Cluster heads handle data fusion tasks, and only send fusion results over the backbone to the base station to reduce data transmission. The cluster head performs the fusion operation periodically [19]. Nodes alternate in performing cluster head tasks to distribute energy consumption evenly. The choice of cluster head is based on the percentage of cluster nodes in the whole network and the number of nodes in the past operation. Assume that for a node n, n is a random choice of a number between 0 and 1, as a flag value. If the flag value of n is less than a threshold *T*(*n*), node n acts as the cluster head node of the current round. The threshold *T(n)* is as follows:

(1) $$T\left(n\right)=\{\begin{array}{c} \frac{P}{1-P*\left(r\;mod\left(\frac{1}{P}\right)\right)},\;if\;n\in G\\ 0\;,otherwise \end{array}$$

where *p* is the percentage of the number of nodes in the network cluster node, *r* is the current number of rounds, and *G* is a set, and the nodes in the set are nodes of the cluster head nodes in the first 1/*p* round. The symbol mod is a modulo operation symbol. Using this threshold, each node can act as a cluster head node in the 1/*p* round. All nodes selected as cluster heads broadcast messages to the network informing the other nodes that they are cluster heads. All non-cluster head nodes receive the broadcast message and decide which cluster to join based on the received signal strength.

LECAH-C is an improved version of LEACH. It uses the base station center control method, and applies an annealing algorithm to get K optimization of the cluster head and the corresponding cluster in the base station. The base station broadcasts the new cluster head IDentity (ID) information to each node to form a clustered network. After the selection stage of the cluster head, the remaining stages in LEACH-C and LEACH are the same, but its performance is better than the LEACH protocol.

##### Chain-Based Routing

**Figure 4:**
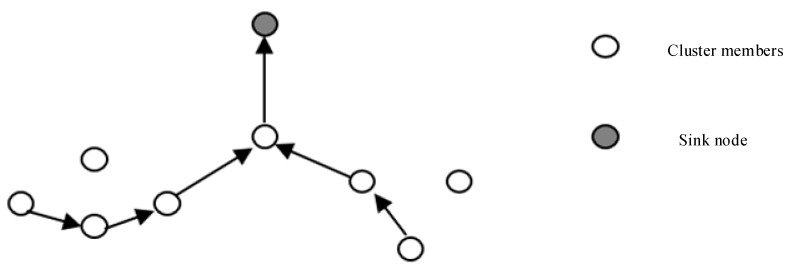
Example of single-chain structure.

Power-Efficient GAthering in Sensor Information Systems (PEGASIS) [20] and its high-order algorithm have improved the fusion method of LEACH, as shown in Figure 4. It is based on two assumptions. The first is that all nodes are far from the sink nodes; the second is that each node can integrate the received data packets into their own data into a packet of the same size. The PEGASIS algorithm collects data before the first use of a greedy algorithm to connect all the nodes in the network into a single chain, and then randomly selects a node as a special node. The special node sends data collection requests, and data from the single and the endpoints flow to the special node. Intermediate nodes perform a data fusion before passing the data, and finally the data passes from the special node to the sink node.

The advantage of this algorithm is that the single-chain structure minimizes the transmission distance of each node, and uses only one node for long-distance data transmission. This makes the algorithm more energy-efficient than the LEACH algorithm, but it does have two shortcomings of greater average delay and poor robustness.

### 2.2. Routing for Mobile Agent

One of the most critical problems to solve in mobile-based applications is the migration path of mobile agents, that is, the choice of routing. For instance, network management may involve frequent operations by multiple mobile agents. Each mobile agent moves through the network on a designated path to collect information for further processing. The agent returns the result to the management station so that it can follow up. If the mobile agent selects a bad traverse order, this affects the operational efficiency, increases the system response time, increases network traffic, and even leads to operational abnormalities.

While advances in sensor technology have made it possible to achieve a “quantity-to-mass” by using large numbers of inexpensive compact sensors in very complex situations, we must apply new computing technologies to meet the theoretical and methodological challenges. Communication requires consumption of limited energy available in sensor nodes, while large numbers of sensors often have different forms of data. Selecting only the most needed information becomes key. Qi [21] proposed mobile agent-based distributed sensor networks distributed to leaf nodes. The purpose reducing network resource consumption (mainly bandwidth) and risk of intentional monitoring by enemies. In this network configuration, the processing unit dispatches mobile agents with executable data synthesis instructions. The mobile agents selectively access leaf node sensors along the specified path and progressively fuse the data.

Sensor nodes, also called leaf nodes, are the basic data collection units in mobile agent routing sensor networks [22]. Sensor nodes have several channels, and multiple sensors connect to them. Base stations send sensor nodes to collect diverse types of measurement data in the external environment, such as sound, vibrating, and infrared. The sampling subsystem controls data acquisition and transfers collected data to the main system for processing. Nodes detect the signals energy in each channel individually and process data at the analog front end. Mobile agents move between sensor nodes in the network, sequentially integrate the local data with the desired resolution, and send the result to the first processing unit. Due to intermittent failures or energy-saving considerations, some sensor nodes may be off or dormant, to be re-awakened when necessary. The sensor finds its longitude and latitude from its Global Position System (GPS) module. The embedded Radio Frequency (RF) modem in sensor nodes reduce network energy requirements [23]. Different clusters select different network members to avoid interference. The message transmission relies not only on the physical distance between two sensor nodes, but also on communication bandwidth, data packet loss rate, and length of the transmitted message (including partially integrated data and mobile proxy code).

**Figure 5:**
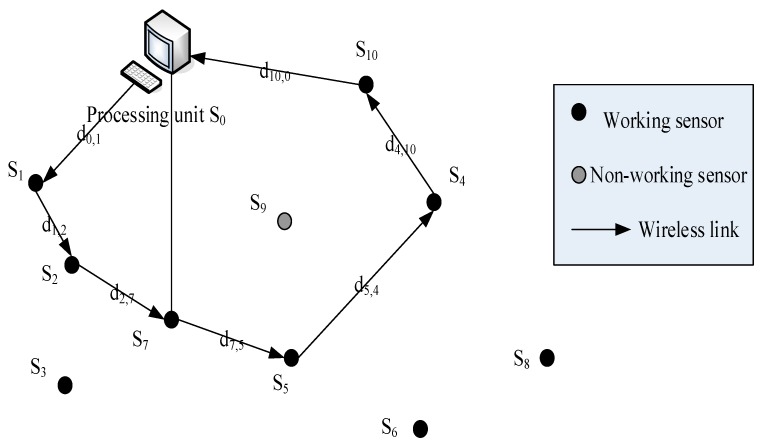
A simple mobile agent sensor network with 1 processing unit and 10 leaf nodes.

Figure 5 shows a simple configuration of a sensor network based on mobile agents. The network has a processing unit, denoted as $s_{0}$, with *N* = 10 leaf nodes, denoted as $s_{j}$, and i=1, 2,…N, one of them closed. The sensor node space travels in a monitoring area, and each node collects measurement data in the environment. The sensor node $s_{j}$ detects the signal energy expressed by $e_{i}$, and i=1, 2,…N. The time taken by the sensor node $s_{i}$ to collect data is $t_{i,acq}$, and the time consumed by the processing data is $t_{i,proc}$. The signal bandwidth of the wireless communication link with distance $d_{i,j}$ between sensor nodes $s_{i}$ and $s_{j}$ is W bit and the operating frequency is $d_{i,j}$ Hz. Due to intermittent errors, some sensor nodes may be temporarily off, such as the sensor $s_{9}$ in the figure. The purpose of the route is to find a path for the mobile agent that satisfies the required detection accuracy with minimal energy consumption and path loss. The energy consumption depends on the working energy and the computation time of the process, and the path loss is directly related to the physical length of the selected path.

Mobile agents dispatched by the processing unit will access sub-sensors in their cluster to fuse the data received in the coverage area. As the number of sensors increases, the use of good data fusion algorithms can improve accuracy. The routing algorithm selects the next best route to get the desired signal energy level based on limited total energy consumption and path loss [24], which can make data analysis better in Wireless Sensor Networks [25].

### 2.3. An Agent-Based Model of Business Networks

Networks and network effects occur in many areas, including business. Recently, Cabral et al. proposed an agent-based network model to analyze the impact of business interoperability on the performance of cooperative industrial networks [14]. In the business world, multiple organizations routinely undertake collaborative temporary projects. The Information and Communication Technology (ICT) platforms are heterogeneous, activities dependent on others’ progress, relationships and communications interdependent, and the composite result highly dependent on all participants. Delays for one can delay whole projects. The authors conducted the study first as a simulation, and then confirmed with a real-world case, as is usual in computer network research. The case was the construction of a dam, the specific application testing of the quality of concrete for the dam, and the network system a middleware system of Radio Frequency Identification (RFI) sensors to ease communication between collaborators. Using the new system, communication of test results improved up to 98% [14]. Table 1 presents some salient similarities between business networks, the Dam project, and mobile wireless sensor networks.

Based on these and other similarities, we developed an equivalent model for mobile wireless sensor networks.

### 2.4. MWSN Architecture

**Figure 6:**
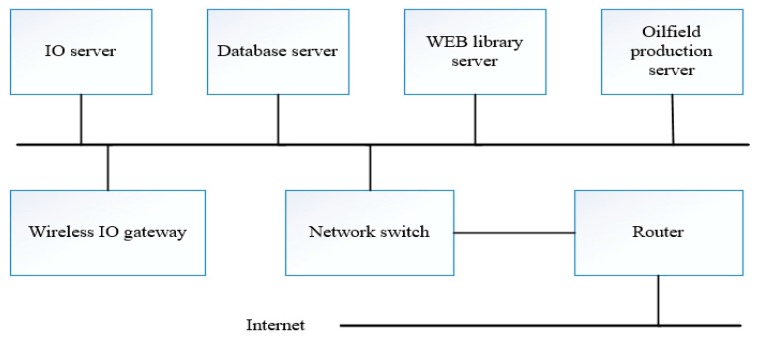
Block diagram of the main node of the scheduling and control center.

When designing networks, we should consider the content of communication, storage and service in addition to considering the node’s self-function. Figure 6 describes the system structure of a typical network suitable for multi-level wireless sensor network design applied to an oilfield production system. The system structure includes a wireless Input Output (IO) gateway server, IO server, database server, WORLD WIDE WEB (WEB) server and oil production server. Network devices can be shared in the control room, but switches and routers need an independent IP address. Most sensors are stationary and connect to equipment, but the network can add to the network as needed.

#### 2.4.1. Distributed MWSN Architecture

In the actual situation of oilfield development, we can use network communication and mobile agent data fusion technology to create a dynamic multimedia WSN system. It carries monitoring data and controls the transmission of multimedia information. Communication energy consumption accounts for a large part of the energy consumption of the entire sensor network due to the large amount of multimedia information. The MWSN has multi-level, multi sink, distributed sensor nodes of a variety of network systems [26]. Moreover, it is necessary to classify monitoring data from different nodes, all of which need many data transfers.

To reduce the frequency of communication, limiting the scope of data communication to local clusters is a better solution. Due to their proximity, nodes in the same cluster often receive identical information. Temperature sensors in adjacent regions often record the same temperature. Avoiding duplication, cluster nodes extract relevant useful information locally, use this information, and communicate it to each other through a broadcasting system [27]. Data replication technology can improve data transmission rates, which can help data recovery [28] quickly. MWSN often have poor connectivity between the mobile sensors. If nodes lose connection and data must send to the next hop node, they store the data temporarily until the connection is re-established or the node finds a better path. Therefore, the sensor nodes need to have a certain storage ability.

**Figure 7:**
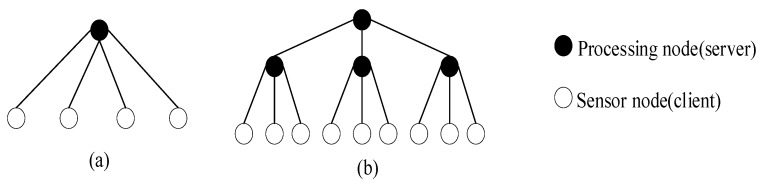
Several client/server models. (**a**) all sensor nodes send data directly to the processing center; (**b**) hierarchical data processing solves transfer problems step by step.

Sensor networks have two types of calculation models for collaborative information processing. Key distinctions are the type of payload (code with processing code or data) and the location of integration (locally or in the processing center). In the traditional client/server computing (C/S) model [16], all sensor nodes send data directly to the processing center as shown in Figure 7a. In applications with substantial amounts of data, sensor nodes usually only compress the data collected and transfer it directly to the processing center for processing. On larger scale, hierarchical data processing solves transfer problems step by step as shown in Figure 7b. These methods are not efficient enough in the case of wireless network with limited bandwidth, where the amount of data is increasing, and the energy of each sensor node is very limited.

Although client/server based distributed computing is popular, it is not suitable for development of sensor networks. Sensor networks have unique independent features in combination with mobility. They have very limited resources and large numbers of sensor nodes. Sensor nodes and communication links are prone to failure, and have abnormal dynamic characteristics. When their energy is exhausted, existing sensor nodes will stop working, and the network must use other mobile sensor nodes.

In the mobile agent computing model, the transfer unit is the mobile agent itself and the final data fused at each sensor node. This model has the following obvious advantages: scalability, reliability, multi-task adaptability, energy-awareness, and accuracy of fusion. With mobile agents, increasing numbers of sensor nodes do not affect performance. Mobile agents set up connections and return results. Designers can give them a variety of software tasks to expand the network functions. Information gain and energy restrictions dynamically determine the path of mobile agents. The accuracy of the fusion results increases with the number of agents. Agents can either visit all nodes on the migration path, or they can avoid unnecessary migration. The former increases accuracy, the latter is more efficient when the amount of data is much larger than the software code.

Peng and Low [29] propose a data centric energy efficient routing protocol by using wireless local area network technology, while Wang et al. propose a multi hop sensor network based on clustering of self-organizing routing mechanisms [30]. An on-demand passive clustering mechanism [31] overcomes the two limitations of the self-organizing routing mechanism, that is, the limited scalability and low adaptability of high-density sensor networks. In addition to routing protocols and network architecture, researchers have extensively studied other aspects of sensor networks. For example, collaborative signal processing uses distributed data compression and transmission [32]. This paper presents special research on an important task in sensor networks, the target detection. There have been many papers and monographs on this subject. We propose optimal fusion rules under the assumption of conditional independence, and study the decision fusion in detail. Specifically, this study presents a solution for optimal sensor node selection under the condition of many communication constraints.

We assume that the performance of local sensing nodes is known. However, it is very difficult to estimate the performance of local sensor nodes through the experiment for a dynamic target and passive capture information sensor nodes. The performance of the target changes in real time with movement, even if the local sensor nodes can estimate their detection performance to a certain extent in real-time, but the cost of transmitting to the fusion center is very large. For most inexpensive sensor networks based on delay tolerance, limited communication and saving energy and bandwidth are very important. In this paper, we propose an information fusion algorithm based on a multi mobile agent framework and statistical decision method to solve the problems above.

### 2.5. Networked Color Sensors

The second part of our research involves the use of color sensors to validate the theoretical model. In this section, we discuss some aspects of color sensors that are relevant to the study.

#### 2.5.1. Color Sensors

Color sensors are one type of many functional sensors with a significant role in daily life. The color of objects hold a lot of information. Many factors, such as radiation light and reflections, light source azimuth, observation orientation and the performance of the sensor, easily affect them [33]. Changing any parameter will lead to change in the observed color. At present, two basic types of sensors exist based on type of color identification:

(1) RGB color sensors (red, green, blue) that detect tristimulus values. Three sensors, one for each color, record the proportions of red, blue, and green. Changing the detection distance changes only the light intensity but not the proportion of the colors. This allows for correct use even on targets with mechanical vibrations.
(2) Spectral sensors with multiple sensors, each sensitive to spectral reflectance at each wavelength or in a narrow wavelength range. These instruments have high accuracy and the ability to measure absolute colors. Research uses these sensors often, but they are sensitive to movement of the target.

#### 2.5.2. Partial Color Detection

Color is a perceived quality. We know that there is a difference between the real color of an object surface, and the color as measured by an imaging device. The surrounding environment influences this partial color, especially the background around an object. The color of the light in which it is perceived, also influences the recorded color. Color temperature [34] of the light source moves more towards blue when the light color temperature is higher, and towards red when the light color temperature is lower. Different light sources like natural light, tungsten filament lamps, halogen lamps, and LED lamps, all have their own light color temperature. These, and the characteristics of imaging devices themselves, influence how we record color.

Before we attempt to restore the recorded color to the real color, we need to know if the image uses only partial color. Some representative partial color detection methods, including histogram statistics [35], gray balance method [36], white balance method, etc. can detect partial color in images. Histogram statistics show the complete color spectrum of the image. They will give the average brightness of three channel of red, green and blue (RGB) color space. We can judge whether the color of the original image is partial by comparing the average brightness of R, G and B channels. If the brightness of any is higher, the whole image color shifts toward that color. This method can detect that the shift exists, but not the reason for it. The Gray balance method assumes that the mean of the R, G, B values is equal in the whole image, which embodies as neutral “ash”. The average brightness of every channel is corrected to Lab color space. The difference between recorded values and Lab values shows if a partial color exists. Light strength in the environment influences this method, as well as singular color in the image. White balance method deals with the existing mirror reflection image. It considers that the specular part of the mirror or the white area reflection can reflect the light color of the light source. We count the highest brightness value of every channel, convert it to Lab color space, obtain the relative homogeneous Lab coordinates, calculate the color lengths to the neutral point, and judge whether there is partial color. The absence of white or specular part in the objects distorts the results.

All three methods are only suitable for some but not all situations. Therefore, average image color and highest brightness value limit partial color detection. We need to develop other methods for partial color detection.

#### 2.5.3. Color Correction

The next step after color cast detection is color correction. Color correction describes object intrinsic color under different lighting conditions, and applies to medical images, remote sensing images, mural images, licenses and many others. Some classic methods for color correction are grey world color correction [37] and perfect reflection color correction [38].

(1) Grey world color correction assumes that in a normal well color balanced image, the average of all colors is a neutral grey. We can calculate the illuminant color by comparing the average floor with neutral grey, but we cannot use this method in images with a single dominant color.
(2) Perfect reflection color correction. The object itself has no color; it shows color through a different wavelength of light absorption, reflection and projection. If the object is white, it reflects all light. The white object or area is the perfect reflector. Perfect reflection theory assumes that the perfect reflector is standard white in the image. No matter which light, a white object, the R, G, and B of its image are great values. Based on the perfect reflector, they correct other colors.

The two-color correction methods are suitable for most color corrections. The calculations are simple, but sometimes cannot restore the real object color. We will use our proposed method to verify the feasibility of the protocol using color sensors.

## 3. Improved Multi Mobile Agent Data Fusion Scheme

### 3.1. Multi Mobile Agent Framework

This section presents the detailed design for the proposed mobile wireless sensor network framework. This approach balances performance with energy consumption and network stability. The mobile agent framework consists of two main components. The first component is the physical architecture, the second component the agent software with the middleware layer.

#### 3.1.1. Physical Architecture

The physical layer can contain a variety of sensor nodes. They can be fixed or mobile, wireless or wired, and use data centric or IP centric routing protocols. Based on sensor function, we use the following categories:

- Sensor nodes (SN): smart sensors, Personal Digital Assistants (PDAs), laptops, Tablet PCs, mobile robots, radio frequency interrupt links etc. which communicate wirelessly.
- Cluster head nodes (CHN): based on the needs of the application, any node may become a cluster head node.
- Regional management stations (RMS): powerful workstations communicating wirelessly with sensor nodes, and directly connected to the wired network that manages the subnet of the sensor network. It is an application defined by a geographic region, like the base station in a conventional wireless network.

#### 3.1.2. Software Architecture and Middleware Components

In this framework, sensor network software consists of two components: sensor node software and regional management station (RMS) software. Each component holds at least an operating system (OS) and middleware. The operating system meets the special application requirements of the sensor nodes. The sensor node software also includes the sensor manager to manage and measure local sensing data. It has interfaces for middleware to access sensor information. RMS software also has data mining, node management facilities, and a specific version of the code (usually the tracking and classification algorithm) for the body of the sensor node based on the requirements of a specific application.

The following middleware delivers distributed hierarchical services for sensor networks. The sensor node middleware coordinates the integration of sensor nodes and other nodes of the network. It consists of the local controller, the management module, the communication module, the mobile module, the special task algorithm, the directory service and other downloadable executable files. It is based on proxy software, and provides the embedded agent platform.

Regional Management Station (RMS) middleware coordinates the integration of sensor nodes and other nodes of the network. It holds the management module, communication module and controller. The middleware is based on the proxy software and provides services for the sensor nodes and software agents.

#### 3.1.3. Application Information Fusion Framework for Sensor Networks

Mobile agent frameworks is very useful for collaborative information processing. They promote the integration of the hardware agent of the physical environment and the application layer of the software agent. The two main characteristics are the agent’s communication and mobility.

The Interoperability agent model [14] shows an information network architecture for business processes. The most important part is the middleware, which provides shared information based on data from RFI technology. In our sensor network framework, we extend the model, and the extended model has the following software components replacing the RFI middleware:

- Sensor Agent (SA): part of the sensor node, and can access the data source of the sensor node. It manages sensor nodes to make information collected by the sensor node available for specific applications.
- Agent Management System (AMS): manages the agent, including registration, authentication, security, and mobility. Extensions of the software can support clustering and cooperation. It also registers Sensor Agents
- Directory Facilitator (DF): delivers query services for the upper application, and finds suitable partners for other agents in a session.
- Agent Communication Channel (ACC): a message routing agent that sends the request message to the sender and supports local and remote communication.
- Data Manager (DM): main task is to manage and tap the local sensing data.
- Application Agent (AA): a static or dynamic proxy that communicates, moves, collaborates, analyzes, adapts, learns, and performs other application specific tasks.

Table 2 shows the data structure of the application agent. It consists of four components: ID number to distinguish it from other agents, Data space to store the results of intermediate integration; Service classes to distinguish between integration algorithms; and Migration route of the mobile agent.

In the above six types of agents, sensing nodes may include SA, AMS, ACC, DF and AA. Regional Management Stations may include ACC, AMS, DM and AA. They support local and remote communication and use wireless transmission to communicate between nodes. Software agents communicate with each other from point to point for local and remote communication, and use wireless transmission as a mechanism for communication between nodes.

### 3.2. Data Fusion Algorithm

We assume that the *N* sensor nodes follow a random distribution in the region of *b*^2^, and that the position of each sensor is independent of each other. This satisfies the uniform distribution:

(2) $$f\left(x_{i},y_{i}\right)=\{\begin{array}{c} \frac{1}{b^{2}},\;-\frac{b}{2}\leq x_{i},y_{i}\leq \frac{b}{2}\\ 0,\;otherwise \end{array}$$

where *i* = 1, ..., *N*, (*x_i_*, *y_i_*) is the coordinate value of sensor node *i*.

We assume that the noise of local sensor nodes follows the standard Gauss distribution:

(3) $$n_{i}∼N\left(0,1\right)$$

For sensor node *i*, the two-element hypothesis detection problem is:

(4) $$H_{1}:s_{i}=a_{i}+n_{i}\;H_{0}:s_{i}=n_{i}$$

where *s_i_* is the received signal, *a_i_* is the signal amplitude.

The signal energy emitted by the target decreases with the increase of distance. We employ an isotropic signal power decay model as follows:

(5) $$a_{i}=\sqrt{\frac{P_{0}}{1+ad_{i}^{n}}}$$

*P*_0_ is the signal power of the target when the distance is 0, *d_i_* is the distance between the target and the local sensor node *i*:

(6) $$d_{i}=\sqrt{{\left(x_{i}-x_{t}\right)}^{2}+{\left(y_{i}-y_{t}\right)}^{2}}$$

(*x_t_*, *y_t_*) are the coordinates of the target, *n* is the signal attenuation index. Its range is (2, 3), *a* is an adjustable constant. The greater the value, the greater the signal attenuation.

We can define a parameter at first:

(7) $${\mathrm{SNR}}_{i}=a_{i}^{2}=\frac{P_{0}}{1+ad_{i}^{n}}$$

When the distance is 0, SNR value is:

(8) $${\mathrm{SNR}}_{0}=10log_{10}^{\left(P_{0}\right)}$$

We assume that all sensor nodes make decisions with the same threshold *τ*, threshold *τ* and false alarm rate *P_fa_* satisfies the following relations:

(9) $$P_{fa}=\int_{\tau }^{\infty }\frac{1}{\sqrt{2\pi }}e^{-\frac{t^{2}}{2}}dt=Q\left(\tau \right)$$

(10) $$\mathrm{Or}\;\tau =Q^{-1}\left(P_{fa}\right)$$

*Q*(·) is a complementary function of the standard Gauss distribution function, that is:

(11) $$Q\left(x\right)=\int_{\tau }^{\infty }\frac{1}{\sqrt{2\pi }}e^{-\frac{t^{2}}{2}}dt$$

The detection probability of the local sensor i is:

(12) $$\begin{array}{cc} P_{di} & =\int_{\tau }^{\infty }\frac{1}{\sqrt{2\pi }}e^{-\frac{{\left(t-a_{i}\right)}^{2}}{2}}dt\\ & =Q\left(\tau -\sqrt{\frac{P_{0}}{1+ad_{i}^{n}}}\right) \end{array}$$

Using binary data *m_i_* = {0,1} to represent sensor nodes, when the value of *m_i_* is 1, it indicates that sensor node *i* has probe event, otherwise the value is 0. All sensor nodes within a cluster are represented as *M* = {*m_i_*: *i* = 1, ..., *N*}, When the mobile agent assigned by AMS collects the data *M*, the fusion center DM makes the final decision with the integration algorithm.

We know that the best decision fusion rule is the Chair-Varshney fusion rule. Its statistical function is:

(13) $$\Lambda_{1}=\overset{N}{\underset{i=1}{\sum }}\left[m_{i}ln\frac{P_{di}}{P_{fai}}+\left(1-m_{i}\right)ln\frac{1-P_{di}}{1-P_{fai}}\right]$$

Each sensor node of the false alarm rate can be (*P_fai_* = *P_fa_*) by Formula, we only need to know the threshold can be τ. However, the detection rate of *P_di_* for each sensor node is very difficult, because the distance between the sensor node and the target and the signal amplitude of the target determine the detection rate. All sensor nodes may directly send collected data to the fusion center DM, and the fusion center makes decisions based on original testing. However, the direct transmission of raw data needs large overhead due to low energy and bandwidth, so the transfer of binary data to the processing center is a very good way to deal with this.

We assume that *P_di_* = *P_d_*, *P_fai_* = *P_fa_*, and *P_d_* > *P_fa_*, the statistical function is simplified as follows:

(14) $$\Lambda_{2}=\overset{N}{\underset{i=1}{\sum }}m_{i}$$

Therefore, the fusion rules of fusion centers are:

(15) Λ2=∑i=1Nmi≃TH0H1

We want to detect the number of mobile agents to collect, and use the total *m_i_* value and the threshold value τ.

The main task of Processing center, including data management for the DM, is data mining and fusion. For data fusion level, we calculate the false alarm rate *P_fa_* as:

(16) $$P_{fa}=Pr\left\{\Lambda_{2}=\overset{N}{\underset{i=1}{\sum }}m_{i}\geq T|H_{0}\right\}$$

Obviously, based on the assumption of *H*_0_, the total number of all detected events $\Lambda =\overset{N}{\underset{i=1}{\sum }}m_{i}$ follows the binomial distribution Binomial (*N*, *P_fa_*). Therefore, for a given threshold *T*, the rate of false alarm is:

(17) $$P_{fa}=\overset{N}{\underset{i=\lceil T\rceil }{\sum }}\left(\begin{array}{c} N\\ 1 \end{array}\right)P_{fa}^{i}{\left(1-P_{fa}\right)}^{N-i}$$

This article focuses on the large sensor networks, so when the number of network nodes is large enough, Equation (17) approximate expression is:

(18) $$P_{fa}\approx Q\left(\frac{T-NP_{fa}}{\sqrt{NP_{fa}\left(1-P_{fa}\right)}}\right)$$

For a given threshold *T*, to get the Receiver Operating Characteristic curve (ROC), false probability as the horizontal axis and the hit probability as the vertical axis form the coordinate chart. The curves of the different results obtained by the different judgment criteria under the specific stimulus conditions need the corresponding *P_d_* value. Due to the different detection rate of different local sensor nodes, the total number of Λ_2_ detection does not follow the binomial distribution under the *H*_1_ assumption, so it is very difficult to derive the analytical expression from the Λ_2_ distribution.

We know that *m_i_* follows the Bernoulli distribution, and the probability of success is *P_di_.* When the sensor node *N* is large enough, {*m*_1_, ..., *m_N_*} is independent of each other under the H_1_ assumption. According to CLT and the distribution of Λ_2_ are close to the Gauss distribution, we calculate the mean *η* and variance $\sigma^{2}$ respectively as:

(19) $$\eta \approx E\left\{\Lambda_{2}|H\right\}=\overset{N}{\underset{i=1}{\sum }}E\left\{I_{i}|H_{1}\right\}=\overset{N}{\underset{i=1}{\sum }}P_{di}$$

(20) $$\sigma^{2}=var\left\{\Lambda_{2}|H_{1}\right\}=\overset{N}{\underset{i=1}{\sum }}var\left\{I_{i}|H_{1}\right\}\;=\overset{N}{\underset{i=1}{\sum }}P_{di}\left(1-P_{di}\right)$$

*P_di_* is a function of [$x_{i},y_{i},x_{t},y_{t}$], in this paper, when *N* is large enough, we can approximate Equation (19) as follows:

(21) $$\eta \left(x_{t},y_{t}\right)=\overset{N}{\underset{i=1}{\sum }}P_{di}\left(x_{i},y_{i},x_{t},y_{t}\right)\;\approx \overset{N}{\underset{i=1}{\sum }}E\left\{P_{di}\left(x_{i},y_{i},x_{t},y_{t}\right)\right\}=N\bar{P_{d}}\left(x_{t},y_{t}\right)$$

Among them:

(22) $$P_{d}\left(x,y,x_{t},y_{t}\right)=Q\left(\tau -\sqrt{\frac{P_{0}}{1+\alpha {\left(\left(x-x_{t}\right)+\left(y-y_{t}\right)\right)}^{\frac{n}{2}}}}\right)$$

Similarly:

(23) $$\sigma^{2}\left(x_{t},y_{t}\right)=N\sigma^{-2}\left(x_{t},y_{t}\right)\;=\frac{N}{b^{2}}\int_{-\frac{b}{2}}^{\frac{b}{2}}\int_{-\frac{b}{2}}^{\frac{b}{2}}(1-P_{d}\left(x,y,x_{t},y_{t}\right))P_{d}\left(x,y,x_{t},y_{t}\right)dxdy$$

Therefore:

(24) $$P_{d}\left(x_{t},y_{t}\right)=Pr\left\{\Lambda_{2}\geq T\right\}\;\approx Q\left(\frac{T-\eta \left(x_{t},y_{t}\right)}{\sigma \left(x_{t},y_{t}\right)}\right)$$

We assume that the target has an even distribution in the ROI, and that the average *P_d_* is:

(25) $$P_{d}=\frac{1}{b^{2}}\int_{-\frac{b}{2}}^{\frac{b}{2}}\int_{-\frac{b}{2}}^{\frac{b}{2}}P_{d}\left(x_{t},y_{t}\right)dx_{t}dy_{t}$$

We make some reasonable assumptions to simplify the calculation, assuming that ROI is very large, the signal energy of the target decreases with the increase of distance (when α takes a large value). Based on this assumption, only a small area of the ROI target near the received signal energy will be significantly greater than 0, therefore, ignoring the boundary effect of ROI, $\bar{P_{d}}$($x_{t},y_{t}$) simplifies to as the value of *x_t_* and *y_t_*.

(26) $$\bar{P_{d}}\left(x_{t},y_{t}\right)\approx \bar{P_{d}}\left(0,0\right)=\frac{1}{b^{2}}\int_{-\frac{b}{2}}^{\frac{b}{2}}\int_{-\frac{b}{2}}^{\frac{b}{2}}P_{d}\left(x,y,0,0\right)dxdy$$

Among them:

(27) $$P_{d}\left(x,y,0,0\right)=Q\left(\tau -\sqrt{\frac{P_{0}}{1+\alpha {\left(x^{2}+y^{2}\right)}^{\frac{n}{2}}}}\right)$$

We can divide Equation (26) into the following two parts, one part is the integration of $\frac{b}{2}$ radius circle, the other part is the integration of the remaining part of the ROI. If we use polar coordinates, the first part will be much simpler:

(28) $$\bar{P_{d}}=\frac{1}{b^{2}}\int_{0}^{2\pi }\int_{0}^{\frac{b}{2}}Q\left(\tau -\sqrt{\frac{p_{0}}{1+\alpha^{n}}}\right)rdrd\theta +\frac{1}{b^{2}}\left(b^{2}-\frac{\pi b^{2}}{4}\right)\gamma \;=\frac{2\pi }{b^{2}}\int_{0}^{\frac{b}{2}}Q\left(\tau -\sqrt{\frac{p_{0}}{1+\alpha^{n}}}\right)rdr+\left(1-\frac{\pi }{4}\right)\gamma$$

The second part of the integration is the integration around the circle, d is very large, and $P_{d}$ is very close to $P_{fa}$. Therefore, it is reasonable to draw the following approximation:

(29) $$P_{d}=Q\left(\tau -\sqrt{\frac{P_{0}}{1+\alpha d^{n}}}\right)\;\approx Q\left(\tau -\sqrt{\frac{P_{0}}{1+\alpha {\left(\frac{\sqrt{2}b}{2}\right)}^{n}}}\right)≅\gamma$$

The approximation is conservative, because d is replaced by a larger number of $\left(\frac{\sqrt{2}b}{2}\right)$, similarly:

(30) $$\sigma^{-2}=\frac{2\pi }{b^{2}}\int_{0}^{\frac{b}{2}}(1-Q\left(\tau -\sqrt{\frac{P_{0}}{1+\alpha^{n}}}\right)\times \;Q\left(\tau -\sqrt{\frac{P_{0}}{1+\alpha^{n}}}\right)rdr+\left(1-\frac{\pi }{4}\right)\gamma \left(1-\gamma \right)$$

Therefore, the detection probability *P_d_* of system level is:

(31) $$P_{d}=Q\left(\frac{T-N\bar{P_{d}}}{\sqrt{N\sigma^{-2}}}\right)$$

The multi-mobile agent framework obtains the fusion results based on target detection probability and false alarm rate function of sensor nodes in the clusters. This paper takes the target classification as an example to introduce the calculation model.

We turn the sensor’s detection rate into a confidence range, where local sensor nodes assign a certain degree of determination to the data processing results of the fusion mobile agent. For example, in a target classification program, the confidence range may be 40~70% that the passing target is a diesel truck, 20~30% that the passing target is an SUV [39].

Multiple models use random distribution of confidence itself. The simplest is the uniform distribution, where the model places the same weight on the self-confidence values of the confidence level, and the Gauss distribution. If the degree of confidence is closer to the center, the weight is greater.

The integrated algorithm must be simple and efficient, because it must meet energy efficiency and real-time response. A satisfactory solution algorithm is the overlap function. Originally proposed by Prasad, and the function is like the histogram function. According to the degree of confidence generated by multi sensor nodes, the number of sensor nodes with the same confidence level combine in the overlap function. The mobile agent at each stop point of the route obtains a partial integration result from the earlier mobile sensor node. These results determine whether the mobile agent needs to continue its mobile route [40].

**Figure 8:**
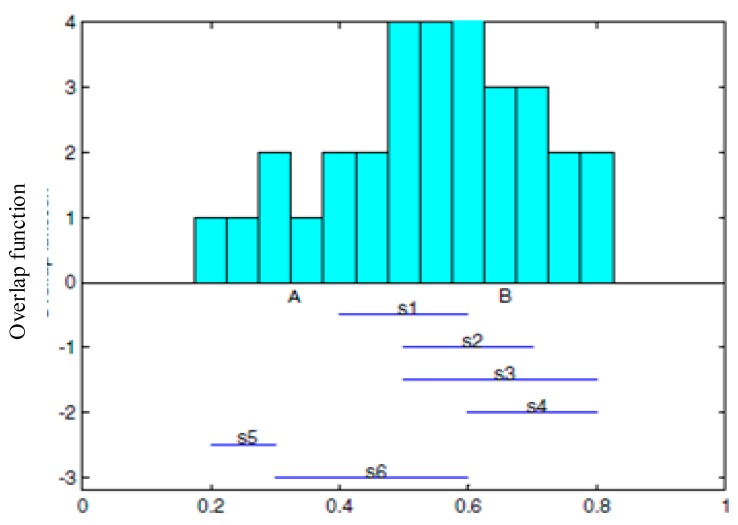
Abstract interval estimation.

The following Figure 8 is an example. It illustrates the structure of overlapping functions of 6 sensor nodes which are uniformly distributed. The horizontal axis stands for the confidence range with a range of 0~1, the vertical axis stands for the number of sensor nodes with the same confidence values. For example, the figure shows that the sensor node confidence range 0.5~0.6 includes s1, s2, s3 and s6.

The integrated information depends on the greatest overlap value [41] in the maximum coverage. For example, as shown in Figure 8, the region [A,B] stands for the integration of information, where the confidence degree of each sensor in this region overlaps most. First, a processing center uses a design overlapping function of generating and analyzing, sends each result to the processing center (RMS) for information integration. In this article, a distributed overlapping function integration is the calculation model based on multiple mobile agents. The integration of overlapping function results in the sensor nodes takes place on the mobile agent migration path. The intermediate result combines with all earlier bounds and the next hop node. The result will be increasingly exact. The converged mobile agent (AA) decides whether the integration results meet the needs of the application, and decide whether to continue to move. The result of the distributed integration is the same as that of centralized processing.

### 3.3. Link Stability in Mobile Environments

In the previous section, the mobile network graph suggests the connectivity concept, which provides the next hop as the migration path of the mobile agent. The choice of the next hop is to find the path of 1- full time connectivity. While implementing this method, we do consider the possibility of setting up a new connection, that is, we consider the stability of newly built link.

The sensor nodes in the sensor network model are mobile, composed of randomly moving sensor nodes and very frequent sink nodes. Due to the limited information dissemination, the communication links of special nodes change often. This change not only affects the ongoing node communication, but also hinders the communication of other nodes in the multi hop network. On the study of stable links in mobile environments, Xiong et al. introduced the empirical distribution of link lifetime and residual link lifetime [42]. Based on these results, they proposed two types of link stability criteria to classify stable links. Lim names continuous effects, but the shortest path of high density in wireless networks tend to be unstable. Other nodes in the communication area edge form the links, and a small movement of any node is enough to destroy the stability.

In studying the link problem in a random mobility model, we assume that there is a link between two nodes at moment t_0_, and use the model to quantify the probability that the link is still valid after time *t*. For independent link failures, we use probability to estimate the reliability of the path after the time *t*. This forms the basis of a dynamic aggregation algorithm, so the network can choose more reliable links to form clusters. However, it may not be realistic to use the standard to select a routing path, because the model is still valid at moment *t*_0+*t*_, even when the link is experiencing one or more faults between $t_{0}$ and time *t*_0+*t*_. The normal approach should be to find an alternative path when a link is in use, instead of waiting indefinitely for the link to return to normal. Jiang overcame this shortcoming by estimating the probability of a continuous link between two nodes in *T_p_* in a period a, where *T_p_* is based on the current activity of the node.

**Figure 9:**
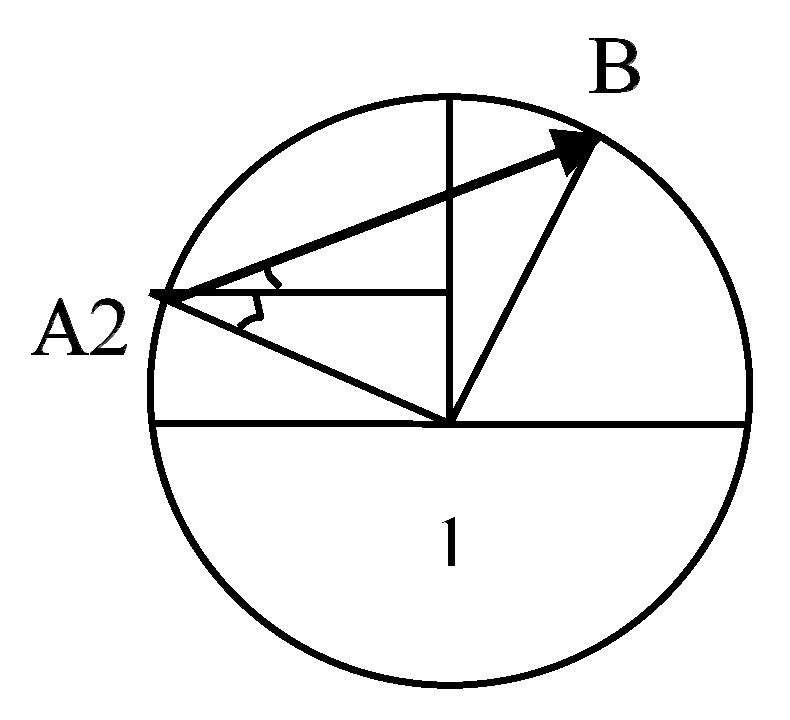
Simulation diagram of node moving radius.

Figure 9 shows the transmission area of node 1, which is based on the node as the center of the circle with radius R, where another node 2 moves from point A to point B along the line AB.

Figure 9 shows the transmission area of node 1. Node 2 enters the region from the A point and leaves the area from the B point. The distance *d_link_* of the node 2 in the transmission area of the node 1 is:

(32) $$d_{link}=\left|2R\cos\left(\alpha +\phi \right)\right|\;=2R\left|\cos\left(\alpha +\phi \right)\right|$$

where *R* is the transmission radius of node 1, which is the angle between the horizontal direction and the direction of point A to node 1, and in turn the angle between the straight-line AB and the horizontal direction.

Link life expectancy is:

(33) $$\bar{T_{link}}\left(v_{1}\right)=\frac{R}{2b}\left(\int_{0}^{\pi }\mathrm{lg}\left|\frac{b+\sqrt{b^{2}-v_{1}^{2}sin^{2}\phi }}{v_{1}+v_{1}\cos\phi }\right|d\phi \right)$$

where $v_{1}$ and *b* are the upper and lower bound of node motion rate.

**Figure 10:**
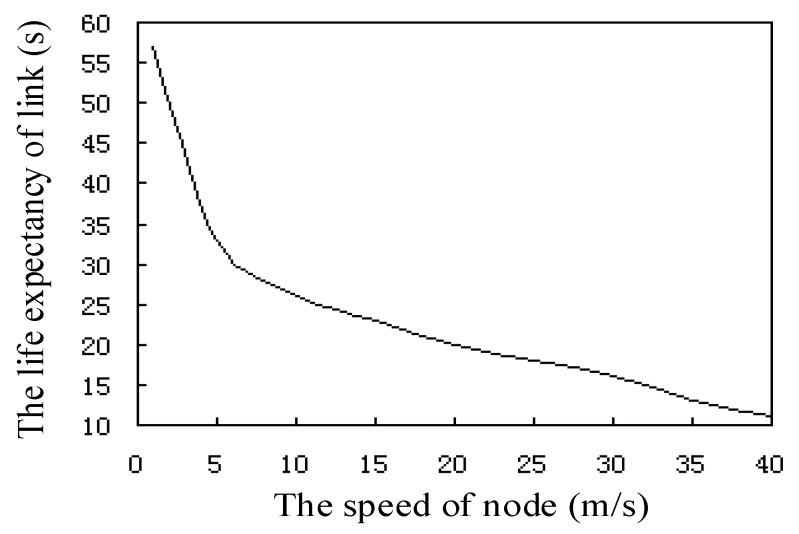
Relationship between the expected life and velocity of the node link (*a* = 0 m/s, *b* = 40 m/s, *R* = 250 m).

Figure 10 shows the relationship between the life expectancy and the speed of the sensor node link, where the speed of nodes in the network is between 0 and 40 m/s. We can see from the curve that the link node life expectancy decreases rapidly with the increase of speed. The time needed for the node to move at 5 m/s speed is two times longer than the time needed for the node to move at a speed of 25 m/s. At the same time, the life expectancy of the link is directly proportional to the node transmission radius R.

For a sensor node moving at a speed of *v*_1_, the cumulative distribution function (CDF) of the link lifetime is:

(34) Flinkv1=1−1π(b−a)∫0π∫02Rtvv2+v12+2vv1cos∅1−(vt2R)2×[u(v2+v12+2vv1cos∅−a)−u(v2+v12+2vv1cos∅−b)]dvd∅

For Equation (34) on the *T* differential, one can get link life proposed distribution function (PDF) $f_{link}^{v_{1}}\left(t\right)$. It is important that network nodes and location distribution do not influence the expression.

**Figure 11:**
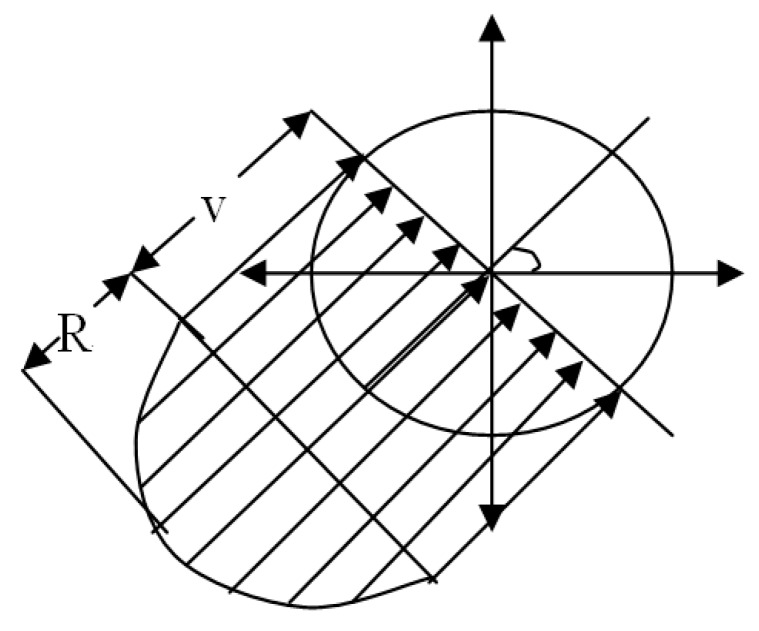
New expected arrival rate model.

In Figure 11, the sensor node 1 is moving at a speed of V relative to the previous static coordinate system XY, and the total expected value of the number of nodes entering the shadow area in *t* seconds is:

E(the number of nodes that will enter the area in T seconds)=η(v)

(35) $$=\frac{2R\sigma l}{\pi \left(b-a\right)}\{b^{2}\varepsilon \left(\frac{v}{b}\right)-2a^{2}\varepsilon \left(\frac{v}{a}\right)+a^{2}\varepsilon \left[\pi -sin^{-1}\left(\frac{a}{v}\right),\frac{v}{a}\right]\;+\frac{v^{2}}{4}\int_{o}^{\pi }\left[1+\cos\left(2\phi \right)\right]\mathrm{lg}\left|\frac{b+\sqrt{b^{2}-v^{2}sin^{2}\phi }}{v+v\cos\phi }\right|d\phi \;-\frac{v^{2}}{4}\int_{\pi -\sin^{-1}a/v}^{\pi }\left[1+3\cos\left(2\phi \right)\right]\mathrm{lg}\left|\frac{a+\sqrt{a^{2}-v^{2}sin^{2}\phi }}{a-\sqrt{a^{2}-v^{2}sin^{2}\phi }}\right|d\phi \}$$

where ε{•} is a complete elliptic integral, ε{•,•} is a non- complete elliptic integral, and Φ is the angle between the direction of movement and the level.

Therefore, when the minimum value of the rate is 0, the expected number of nodes per second into the transfer area is simplified to:

(36) $$\bar{\eta }\left(v\right)=\frac{2R\sigma l}{\pi b}\{b^{2}\varepsilon \left(\frac{v}{b}\right)\;+\frac{v^{2}}{4}\int_{0}^{\pi }\left[1+3\cos\left(2\phi \right)\right]\mathrm{lg}\left|\frac{b+\sqrt{b^{2}-v^{2}sin^{2}\phi }}{v+v\cos\phi }\right|d\phi \}$$

where $\sigma =\rho /\pi R^{2}$ node /m^2^, ρ stands for the average node in a transmission region. The new link to node expected arrival rate is proportional to the average density of nodes in the network transmission and proportional to the radius *R* of the node.

In the above Figure 11, for a given *v*, the probability that cross link arrival time is greater than *t* is equal to the probability of the presence of at least one rate for *v* nodes in the shaded area. Thus, the cumulative distribution function of new cross link arrival time can is:

(37) $$F_{arrival}^{v_{1}}\left(t\right)=1-\frac{1}{\pi \left(b-a\right)}\;\int_{0}^{\pi }\int_{0}^{\infty }e^{-2R\sigma lv}\frac{v}{\sqrt{v^{2}+v_{1}{}^{2}+2vv_{1}\cos\emptyset }}\;*[u\left(\sqrt{v^{2}+v_{1}{}^{2}+2vv_{1}\cos\emptyset }-a\right)\;-u\left(\sqrt{v^{2}+v_{1}{}^{2}+2vv_{1}\cos\emptyset }-b\right)]dvd\emptyset$$

New link cross arrival time for PDF $f_{arrival}^{v1}\left(t\right)$ is:

(38) $$f_{arrival}^{v_{1}}=\frac{d}{dt}F_{arrival}^{v_{1}}\left(t\right)=\frac{2R\sigma }{\pi \left(b-a\right)}\;\int_{0}^{\pi }\int_{0}^{\infty }e^{-2R\sigma lv}\frac{v}{\sqrt{v^{2}+v_{1}{}^{2}+2vv_{1}\cos\emptyset }}\;*[u\left(\sqrt{v^{2}+v_{1}{}^{2}+2vv_{1}\cos\emptyset }-a\right)\;-u\left(\sqrt{v^{2}+v_{1}{}^{2}+2vv_{1}\cos\emptyset }-b\right)]dvd\emptyset$$

We infer that the PDF curve of the new cross arrival time decreases rapidly with the increase of time *t*.

**Figure 12:**
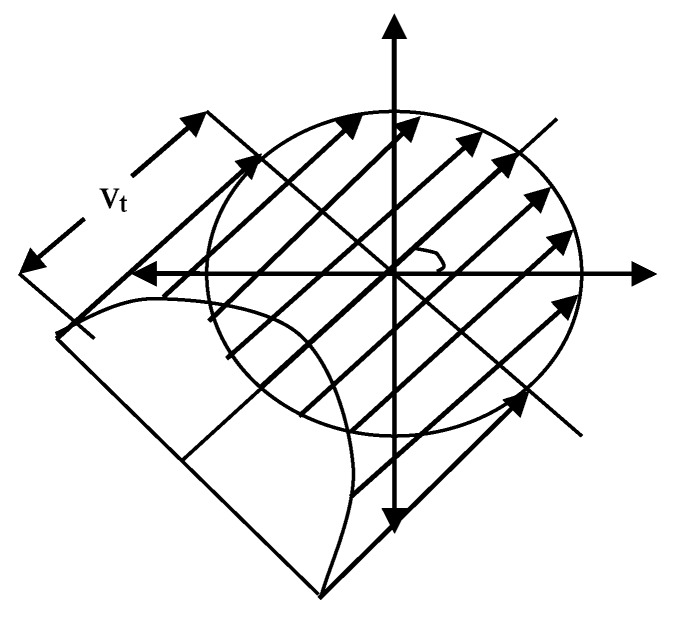
Distributed computing model of link break arrival time.

Using the Figure 12, we calculate the distribution of the interrupt arrival time of the link. The link interrupt cross arrival time for CDF is:

$$F_{break}^{v_{1}}\left(t\right)=P\{\mathrm{Arrival}\;\mathrm{time}\;\mathrm{of}\;\mathrm{link}\;\mathrm{interrupt}\;\mathrm{crossing}\leq t\}\;=\iint^{\text{​}}\left(1-e-2R\sigma lv\right)f\left(v,\phi \right)dvd\phi$$

(39) $$=1-\frac{1}{\pi \left(b-a\right)}\;\int_{0}^{\pi }\int_{0}^{\infty }e^{-2R\sigma lv}\frac{v}{\sqrt{v^{2}+vv_{1}{}^{2}+2vv_{1}\cos\emptyset }}\;*[u\left(\sqrt{v^{2}+v_{1}{}^{2}+2vv_{1}\cos\emptyset }-a\right)\;-u\left(\sqrt{v^{2}+v_{1}{}^{2}+2vv_{1}\cos\emptyset }-b\right)]dvd\emptyset$$

The distribution of the link interrupt arrival time and the cross-arrival time of the new link is the same. We can use the distribution of link lifetime to test the stability of mobile sensor networks. Once a link starts to communicate (here mainly for a single hop migration of mobile agents), its remaining lifetime distribution can be a function of the link lifetime distribution. If the link already exists for *T* seconds, we can express the probability density function of the remaining link lifetime as follows:

(40) $$r_{T}^{v_{1}}\left(t\right)=\frac{f_{link}^{v_{1}}\left(t+T\right)}{1-F_{link}^{v_{1}}\left(T\right)}$$

where $f_{link}^{v1}\left(•\right)$ and $F_{link}^{v1}\left(•\right)$ are the link lifetime PDF and CDF. We apply the link probability density function to the mobile agent migration path algorithm in the next section.

### 3.4. Mobile Agent Migration Path Algorithm

A mobile agent has four characteristics: identification, route, processing code, and memory. The identification registers identity on the network. The route can be pre-determined or adjusted to the network condition. The agent executes processing code locally and uses it both for routing and data collection. The code is adaptable to the task, and the sensor be promoted or demoted as needed. The agent uses memory to store raw data and fused data, depending on the state of integration.

**Figure 13:**
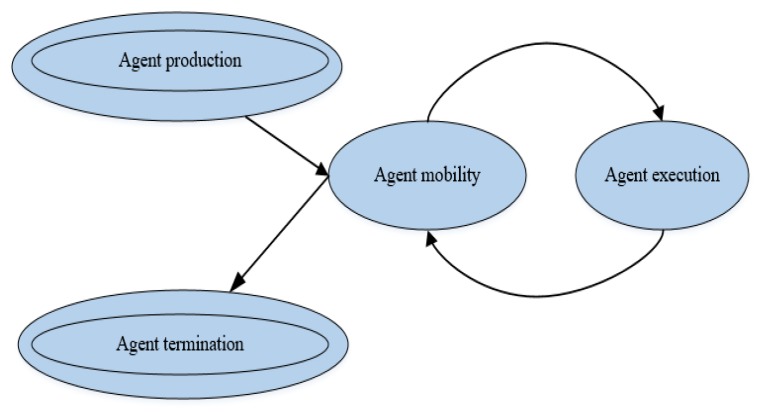
State transition diagram of mobile agent.

Agents can move either on mobile sensors, or the software can move wirelessly from stationary sensor to stationary sensor. In the latter, the source node starts the transmission process, the agent suspends all data collection and starts processing code, the sensor transfers the state information of the agent to the new node according to the routing protocol, and the agent resumes processing at the new sensor. Nodes send results if they are the last hop in the migration route, or if the accuracy of the results meets the required accuracy. After visiting each node, the agent either returns to the host or terminates itself. Throughout the process, service provider (AMS and DF) updates trigger the migration path from source node to destination node [43]. Figure 13 shows the process from agent production, the cycle of data collection and movement, to agent termination.

The choice between static and dynamic routing affects computing time and energy consumption. Dynamic route planning is flexible and adapts to the change of environment in real time [44]. However, it increases computing time and energy consumption. Static routing may not adapt to the changes of the network environment, but saves processing and energy, and calculates static routes only once. Time, energy efficiency, and flexibility are mutually exclusive. Selection of the best path also depends on the number of sensors, which is helpful for node scheduling [45]. With large numbers of sensors deployed, data redundancy can give error tolerance and allow the network to stop collecting data as soon as the fused data has met the specific requirements of the task. It is no longer necessary to visit the remaining nodes in the network.

In this paper, we use a simple sub optimal dynamic path generation method to determine the next hop node in real-time. It considers three parameters: residual energy in candidate nodes for the next hop, energy needed for the next hop, and the geographical distance between current node and potential next node. With identical packet size, communication overhead is proportional to the distance between source and destination nodes. The choice of node for the next hop is the key point. We discussed the different software agents in Section 3.1.3. The main purpose of the directory facilitator (DF) is exchanging information between neighboring nodes through the agent communication channel. This includes residual energy, signal energy, and positional information. It communicates changes in the network promptly. Table 3 shows the information format for the DF exchange.

Our network has two functional levels of agents: data collection agents and managerial agents. We will not discuss the clustering method in the network, but each cluster as an RMS, a DM, and other intermediate components which communicate directly with each other. When sensors detect an event in their monitoring area, they automatically start to collect data. As soon as an amount of data crosses the threshold, sensors send a notification packet to the RMS. The format of the data packet shows in Table 4. Each sensor sends notification packets independently. Upon receipt of notification packets, the RMS checks the source node ID and the geographical coordinates. If the RMS receives more than one packet from the same source ID, it saves the most recent packet and discards the others. Geographical coordinates determine spatial data for the event, and the RMS generates and assigns a mobile probe dect_agent. The mobile agent accesses sensor nodes per the migration route, using the 4.2.4 algorithm to determine whether the sensor node can detect the event. It uses M = {*m*_1_, *m*_2_, ...., *m_n_*} binary numbers (0 or 1) to mark the corresponding results. Finally, the result set moves back to the RMS. The RMS calculates the number of detected events in the result set from the detection function, where a number of *m_i_* = 1 indicates passing the threshold (see previous section). If the threshold is not reached, the detection of mobile agents continues to collect new results, and the cycle continues until it reaches the threshold of event detection or a small value indicates that there is no real detection event. Upon reaching the threshold, the RMS sends a mobile agent fusion_agent, which automatically moves along the migration route from one node to node for data fusion, sending back integrated fusion results. It is worth noting that the migration routes of dect_agent and fusion_agent are the same, but fusion_agent comes after dect_agent. The fusion_agent proxy returns the result to the DM.

Until the fusion_agent data crosses the threshold, sensor nodes continue to collect data until reaching the accuracy of the task or a new round of detection starts. Data formats for the two agents are shown in Table 5.

**Figure 14:**
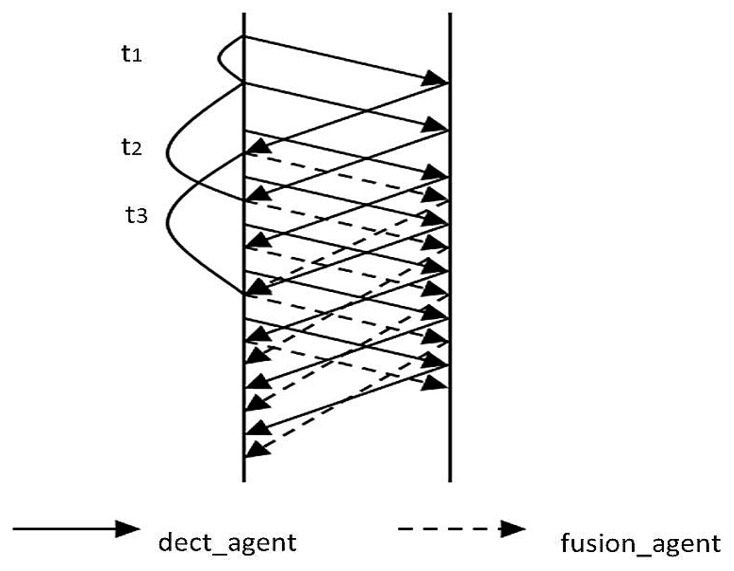
Multi mobile agent working time chart.

Further, consider that new rounds of integration do not start when the current round has not finished. In our design, rounds of integration start periodically and automatically by assigning another fusion_agent to conduct fusion operations. If the number of packets received crosses a certain number, the next dect_agent is automatically generated, even if the first dect_agent has not finished its migration path and the DM has not received its data yet. Given this, data collection by dect_agents is a continuous process. While dect_agents are at rest waiting for a time slice, fusion_agents start working and the two agents alternate with each other as shown in Figure 14. In the figure, *t*_1_ is the time between sending of dect_agents, *t*_2_ is the time dect_agents need to be sent out and traverse the route, and *t*_3_ is the time from sending out a fusion_agent until it returns with its fused data. Since data fusion by the fusion_agent takes more time than detecting an event by the dect_agent, *t*_3_ will be longer than *t*_2_.

When the final integration results meet the accuracy of the task requirements, the two agents will terminate, and the resource recovered. Through direct communication between DM and RMS, the RMS encapsulates data and interacts with the application. Table 6 shows a summary of the complete cycle. The process starts as a linear process of detection and notification of the RMS, followed by a cycle of data collection and fusion, ending in termination of the event and discontinuation of data collection.

**Figure 15:**
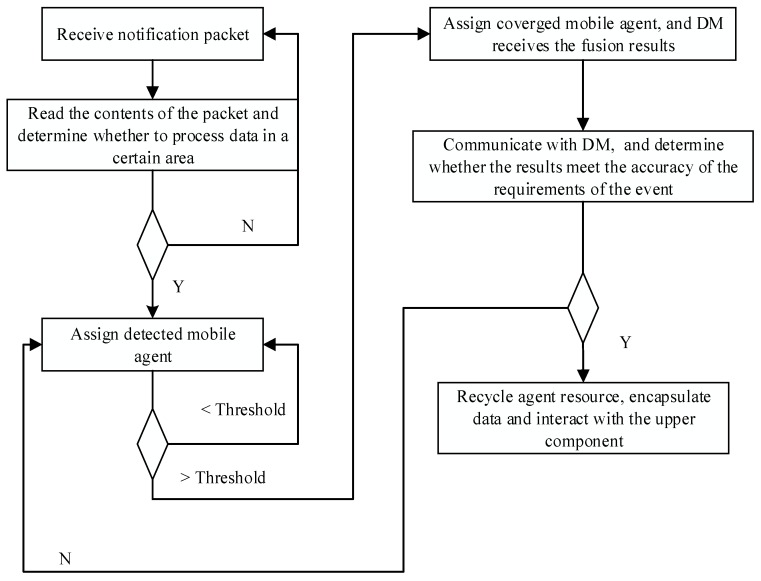
RMS middleware action flow chart.

Cluster heads in the network contain RMS middleware following the workflow as shown in Figure 15 below. One of the key points is the determination of the next hop node. As discussed in the previous section, we can use the residual link lifetime density function to estimate the life of a link in the network. When the mobile agent migrates out of the range of a node into the region of the next hop node, the agent disconnects the old link and forms a new link to the next node. 

If the mobile agent leaves the current node into the next hop node, the possibility of setting up links with all nodes in the next area is *X*1, *X*2, *X*3 *… XK*. The random variable *Y* is the life of the route formed by the *K* links, so the next hop can be found by the maximum of all lifetime links, expressed as follows:

(41) Y=max(X1,X2,X3,⋯XK)

Since all sensor nodes are independent, the life of the new links is independent and identically distributed. In the dynamic sensor network environment, we use random selection for the next hop to adapt to changes in the network topology.

### 3.5. Key Performance Metrics

Although the mobile agent based model has many advantages over the client/server based model, the performance is not always the best. Due to overhead for generation and movement of mobile agents, the performance of different computer models also depends on other parameters related to network configuration. We focus on two criteria for evaluating the performance of these network models: execution time and energy consumption [46].

Execution time refers to the time between starting a task and the user receiving the result. It consists of three parts: *t_trans_* is the time to send data and receive data, *t_oh_* is the overhead time, and *t_proc_* is the processing time. These times are affected to behave different by some factors, such as network transmission rate *v_n_*, data processing speed *v_d_*, the size of the packet $s_{f}$, size of the mobile agent $s_{a}$, message access overhead $o_{f}$ (read data, analyze data, generate and write messages), the mobile agent cost $o_{a}$, the number of *P* nodes, and the number of *m* mobile agents. Each mobile agent may access the number of sensor nodes.

Therefore, we can express the execution time based on the C/S model [47] as follows:

(42) $$t_{cs}=mns_{f}/v_{n}+2mno_{f}+mns_{f}/v_{d}$$

where the data transfer time *t_trans_* = $mns_{f}/v_{n}$, overhead time *t_oh_* = 2*mn*$o_{f}$, and data processing time *t_proc_* = $mns_{f}/v_{d}$.

We can express the execution time of the computation model based on mobile agent with the following Formula:

(43) $$t_{ma}=(m+n)s_{a}/v_{n}+2\left(m+n\right)o_{a}\;+(m+n)s_{a}/v_{d}$$

The proxy transfer time t_trans_ = (*m* + *n*) $s_{a}$/*v_n_*, *n*$s_{a}$/*v_n_* represents the time at which m mobile agents migrate between n sensor nodes. The mobile agent overhead time *ms_a_/v_n_* means that the processing center receives the extra time of the mobile agent to access the sensing node on all migration paths. The proxy overhead time *t_oh_* = 2(*m* + *n*) $o_{a}$, where 2*moa* is the time for AMS to allocate and receive *m* mobile agents, and 2*n*$o_{a}$ is the time for all local nodes to send and receive each mobile agent. The local code processing time is *t_proc_*= (*m + n*) $s_{a}$*/*$v_{d}$.

Energy consumption refers to the total energy consumption of the entire implementation process. Like the rule of execution time, the two models of energy consumption depend on three factors: the energy consumption *e_trans_* of data transmission, processing overhead energy consumption *e_oh_* and the energy consumption *e_proc_* of data processing. For the C/S model and the model based on mobile agent, the energy consumption of the whole data processing of the sensor network is the same in the processing center and local sensor nodes. Therefore, we do not consider *e_proc_* and *e_oh_*, and calculate energy consumption as follows:

(44) $$e_{cs}=e_{tran-cs}+2mnP_{proc}o_{f}$$

(45) $$e_{ma}=e_{tran-ma}+2m\left(n+1\right)P_{proc}o_{a}$$

where *P_proc_* is the energy consumption of the processor under full load.

In summarizing the previous simulation results, we conclude that the model based on mobile agent is not always better than the C/S model, and that different computing styles are only applicable to a particular network configuration.

The computation based on a C/S model is better for few nodes or for limited raw data. The computation based on mobile agents is more suitable for a larger number of nodes, where the number of sensor nodes in the network may reach hundreds or even thousands. In this situation, reliability of links and effective bandwidth decreases, but the model based on mobile agents has higher energy efficiency and execution time. Then again, when the number of sensor nodes in the network is small, the model based on mobile agents will have a larger delay. Based on this, we summarize the following conclusions:

(1) When the number of sensor node is greater than or equal to 17, the computation model based on mobile agents has less execution time and energy consumption.
(2) The optimal number of mobile agents for shortest execution time and energy consumption is usually 5.
(3) When the raw data of the message is much larger than the mobile agent itself, the computational model based on mobile agents has better energy efficiency.
(4) When the cost of accessing files is much larger than that of the agent, the computational model based on mobile agent is faster and more efficient.

In this article, we refer to the above model for performance measurement and simulation. We describe the specific simulation environment and conditions in detail in the next section.

### 3.6. Summary

This section discussed the detailed design for the proposed mobile wireless sensor network. The mobile agent framework consists of two main components: the physical architecture and the agent software with the middleware layer. The physical architecture consists of nodes with distinct functions, and the regional management stations plays a central role in regulating the data collection process through the cluster head nodes. Routing is decided based on three factors: residual energy in candidate nodes for the next hop, distance of the hop, and energy needed for the hop. In the next section, we discuss our use of a network simulation to test energy consumption and execution time between three algorithms, followed by a physical experiment to test the feasibility of data fusion to report sensor data.

## 4. Experiment and Result Analysis

### 4.1. Experiment Environment

We use *NS*-2 network simulation software to simulate the sensor network [48]. *NS*-2 is the most popular event simulator for network research. For simplicity, we assume that there are no two simultaneous events in the sensor field. If an event does occur, all sensor nodes can detect the event and collect original data [46].

The system we use is as follows: the task of the network is vehicle classification, we use a 100 × 100 m^2^ sensor area, the sensor network has 16 clusters, and the adjacent nodes’ spacing of the same cluster do not exceed the length of *d* (500 m). Nodes connect to other nodes in a random model, select a random destination, move at a speed of 10 m/s, send data at 2 Mb/s, and process data at 100 Mb/s. Communication uses the computational model for performance metrics in the third section. Energy consumption of the communication process is proportional to the size of the packet and the square of the distance between the two points. Data fusion reduces the size of data packets by reducing the data correlation. To simulate network mobility for analysis, we make the following assumptions:

1. Nodes use two-way communication links within R meters, and the link will disconnect when one node moves to R meters or beyond.
2. Sensor nodes move at a non-constant speed in a range of [a,b] m/s.
3. The velocity direction of sensor nodes has an even distribution between 0 and 2π.
4. The speed, the direction of motion and position of the sensor nodes are independent of each other. There is no relation between them.
5. A two-dimensional Poisson process with an intensity of σ simulates the location of the nodes in the network. The probability distribution of network area D with *k* nodes around *A* is:
  (46) $$Prob\left(k\;\mathrm{nodes}\;\mathrm{in}\;D\right)=\frac{{\left(\sigma A\right)}^{k}e^{-\sigma A}}{k!}$$

Assumption 1 shows that the signal to interference ratio (SIR) is still high within a certain distance from the target R. The SIR is the ratio between signal strength of the node signal, and the strength of co-channel interference from other nodes within the same area. Close by, sensors receive exact signals. However, as the distance from R increases, SIR will decline, and the bit error rate will rapidly increase to unacceptable levels. Signals may also meet blind areas and multipath attenuation, so the actual transmission area is not fully symmetric. If all nodes in the network use the same transmission power, the network is still approximately homogenous.

Assumption 2 simulates a moving environment where nodes move at different speeds. In our simulation, we focused on a range of high node speeds because the shorter time within range and the challenges of connecting between fast-moving nodes makes it easier to find the worst cases of link characteristics in the background.

Assumptions 3 and 4 describe the aggregation behavior of nodes in large sensor networks. Any association between nodes is unimportant due to the considerable number of independent sensing nodes. Some nodes may collect data from similar targets and move together, but enough data from autonomous nodes models a stochastic process through its composite effect.

Assumption 5 uses the Poisson process to simulate total randomness, which reflects the randomness of the aggregate behavior of nodes in large sensor networks. Equation (46) shows that the number of sensor nodes in the network area *D* is equal to σA. Thus, σ  stands for the average density of network nodes.

In this section, simulation experiments discuss the performance of the proposed algorithm. We compare the algorithm with the client/server model, where nodes send compressed data to the processing center, and another routing algorithm based on best available hops, the Local Closest First (LCF) algorithm. We discussed the C/S model has been discussed in Section 2.4.1. In the LCF algorithm, each mobile agent starts its route from the sink node and searches for the next node with the shortest distance to its current location. Comparing the C/S model and the LCF algorithm [49], the C/S model is a more traditional network model, while the LCF model has better network performance and does not depend on the objective function and coding strategy.

### 4.2. Results and Analysis

**Figure 16:**
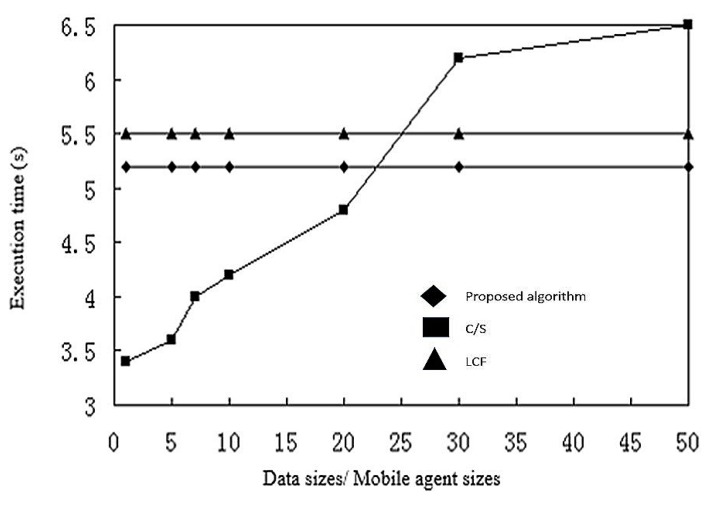
Execution time.

In the simulation, we used as independent variables the size of data and mobile agents, target numbers, and number of nodes in the network. Dependent variables were execution time and energy consumption. In Figure 16, we have a fixed number of sensor nodes, the mobile agent has a fixed size of 1 kb and the data size changes from 1 kb to 50 kb. Since the proposed algorithm and LCF (local search) are based on mobile agents, and mobile agents do not change size, the ratio of data size and mobile agent size has no effect on execution time. We do see that with transmission of data over 23 kb, in terms of execution time, the C/S model performs worse than the mobile agent algorithms (LCF and proposed). The proposed algorithm is marginally faster than the LCF algorithm.

**Figure 17:**
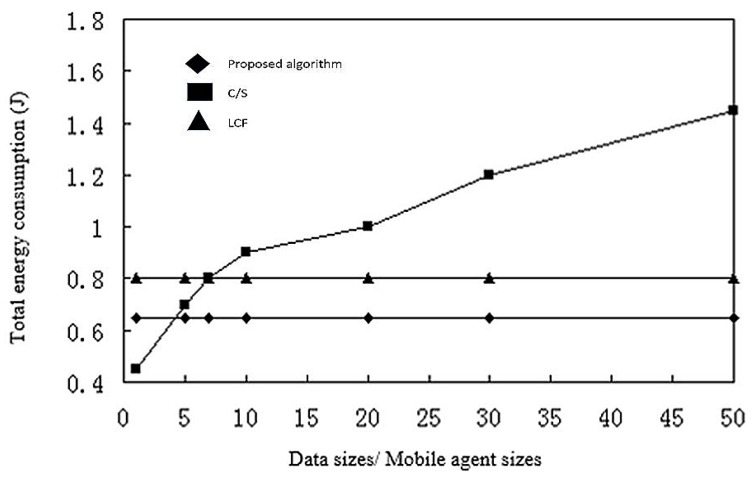
Energy consumption.

The same is true for energy consumption, as shown in Figure 17. Again, the C/S model quickly slows down with data size, and the proposed algorithm is slightly faster than the LCF algorithm. 

**Figure 18:**
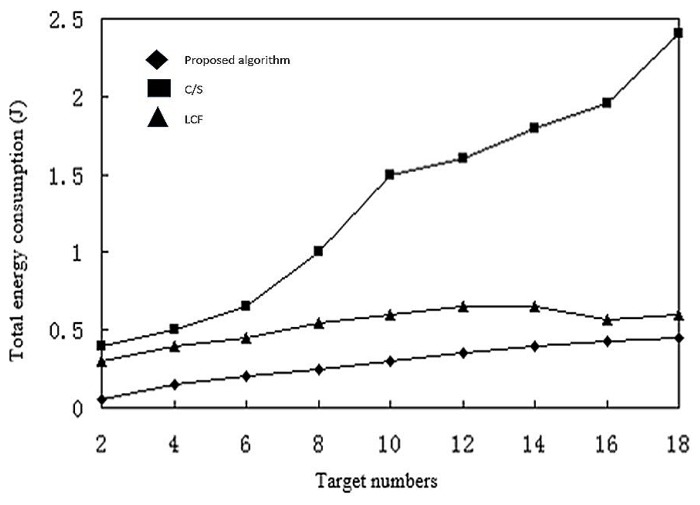
Energy consumption for different target numbers.

Figure 18 shows the network deployment under increasing number of targets. The amount of data for processing also increases, and therefore the energy consumption. The C/S model has a significant increase in energy consumption as the number of targets increases, and the LCF and proposed algorithms are more efficient. Between the two, the proposed algorithm still uses less energy than LCF.

**Figure 19:**
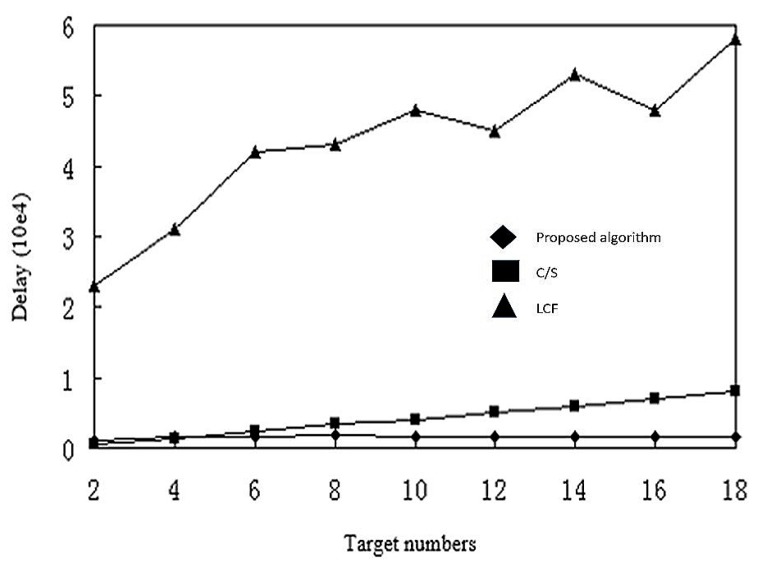
Delay for different target numbers.

Figure 19 shows the network delay with increasing number of targets. The LCF algorithm has the largest delay overall. The proposed algorithm and C/S have similar delays, with C/S slightly slower. In the C/S model, agents transmit the data collected by the sensor nodes around the target, which decreases delay time. The amount of data aggregation to the processing nodes increases with the number of targets. At some point, the processing node in the C/S model becomes the bottleneck, and the data delay increases. The algorithms based on mobile agents have a small extra overhead for small numbers of targets. When the volume of data increases, multi mobile agents migrate constantly between sensor nodes, and transmit only the final fusion results, so the fused data is smaller, and the delay time is shorter.

**Figure 20:**
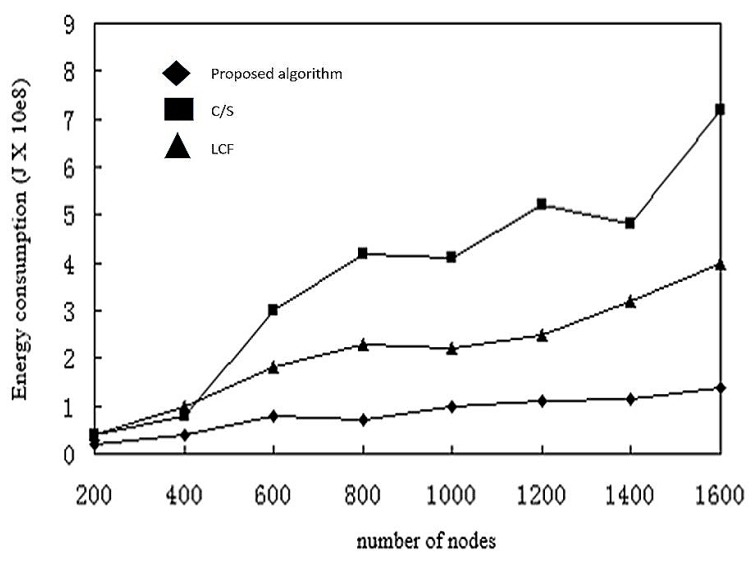
Effect of node density on energy consumption.

In Figure 20, we increase the number of nodes and measure energy consumption. When the node density is small, the node distribution is wider, the correlation between nodes is smaller, and data fusion to reduce the amount of data has less effect. The transmission energy gain cannot offset the energy consumed by the mobile agent itself. With an increase of the number of nodes, the correlation between the data improves, and the energy gain of the mobile agent is much higher than the energy consumed by itself. The energy consumption based on the C/S model is obviously higher than that of the mobile agent. Transmission of raw data uses most energy here.

**Figure 21:**
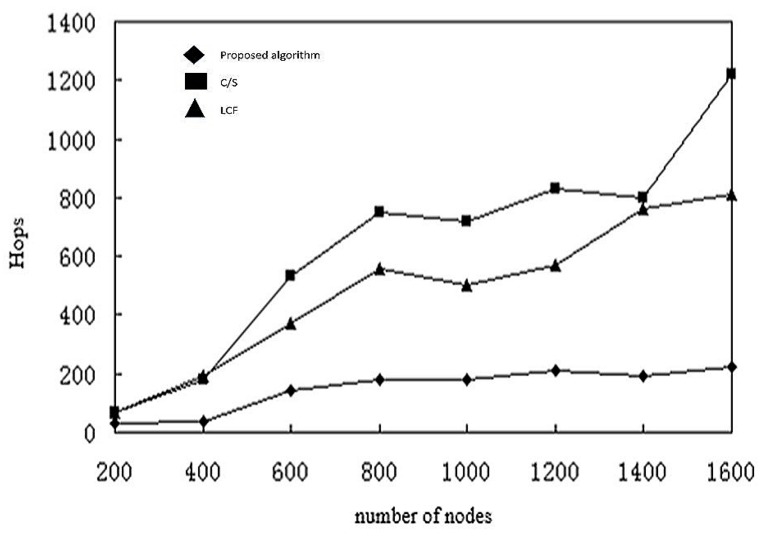
Effect of node density on number of hops.

In Figure 21, we measure the number of hops as the number of nodes increases. The graphs are like the graph showing energy consumption, since transmissions consume most energy. Since the C/S model transfers non-fused data, the total number of transmissions is much larger. Between the two agents, the proposed algorithm has fewer transmissions, since it stops collecting data from the sensor nodes once the threshold has been reached.

**Figure 22:**
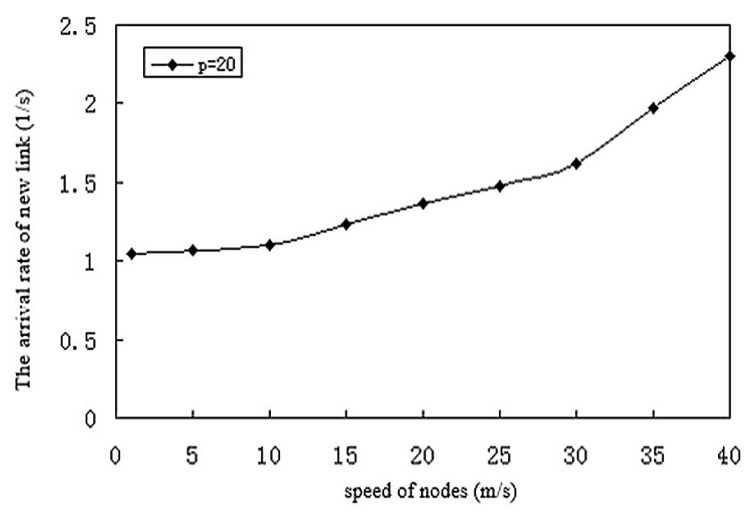
The expected arrival rate of nodes with different speeds.

Figure 22 shows the increase in number of links as nodes move faster and move in and out of range. From Section 3.3, the new link expected arrival rate is proportional to the density of nodes in the network, and the energy of the signal transmission radius of sensor nodes is proportional to the node, P represents the average number of nodes in a transmission area, the value of each parameter is the minimum speed of 0 m/s, the maximum speed of 40 m/s, the transmission radius R of 250 m.

### 4.3. Application Analysis

**Figure 23:**
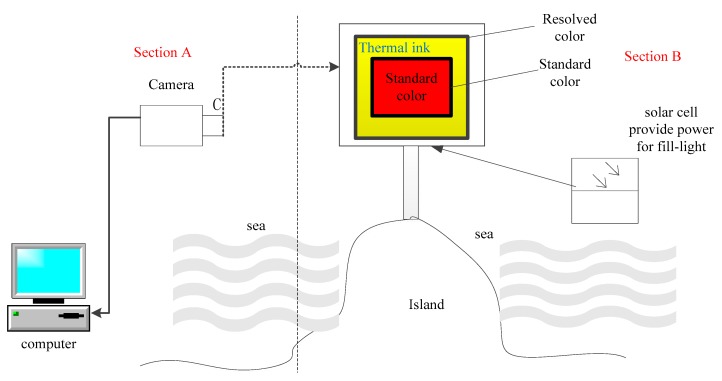
An application of color sensor.

Now that the proposed algorithm showed favorable results for energy consumption and execution time, we also conducted a physical experiment using color sensors to verify the feasibility of using fusion to generate accurate results. As shown in Figure 23, a computer processes and stores images. The camera takes pictures. Section B is the shooting area in the form of a template coated with pigment. Thermal ink measures the color temperature for every color value.

**Figure 24:**
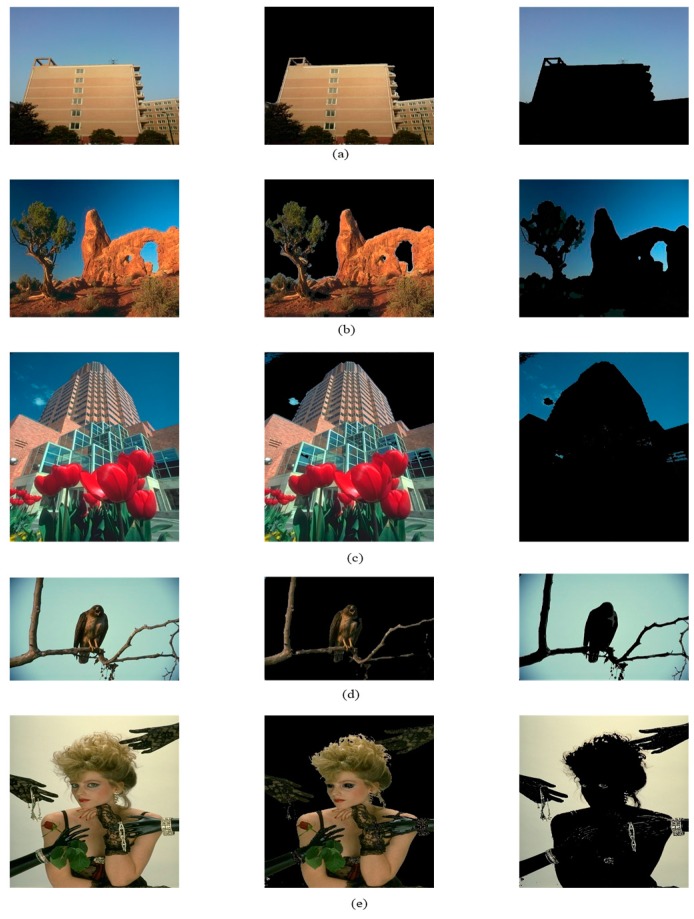
Image fusion results.

Figure 24 shows the image fusion process. From the experimental results, these five sets of images can well separate foreground and background in accordance with the proposed algorithm. In Figure 24a,b, the foreground has a lot of colors, the background is sky. Though in Figure 24a a small portion of foreground is divided into the background, but it doesn’t affect the segmentation results. Figure 24c,d have a little problem with the foreground, cloud is divided into the foreground by mistake. In Figure 24e, the background is similarity to the hair of the woman, and there is a lot black. Hair is divided into the foreground well and black is keep. For each row, the image on the left is the original image, the image in the middle is the foreground part of the image, and the image on the right is the background of the image. The colored section in the foreground and the background are the parts we want. The images show on a screen as in Figure 23, where an area of thermal ink that changes with the surrounding light surrounds an area of standard color. The camera takes long-distance pictures and transfers them to the image acquisition card. The proposed algorithm processes the images and sends them to the computer. The fragmented area holds only thermal ink and standard color, and can detect color value. After color correction of thermal ink color and standard color (since light affects color), we finally obtain the correct color value. We can obtain the temperature value by relating the thermal ink color value and color temperature.

Before data collection, we also considered other influences—day and night, cloudy and sunshine, for example. They change the image appearance. We have put forward a measure for this problem.

We use a high-resolution camera with image stabilization to reduce any added noise effect in the images. The acquisition card is a device for image storage and transmission, and it must have stability and high memory. First, the images need to be pre-preprocessed, where we reduce the influence of noise by using the median filter method. Second, we separate the images with the proposed algorithm. The result is a measuring area and we detect the two-color values in the split images. Finally, we get the precise color value after color correction.

**Figure 25:**
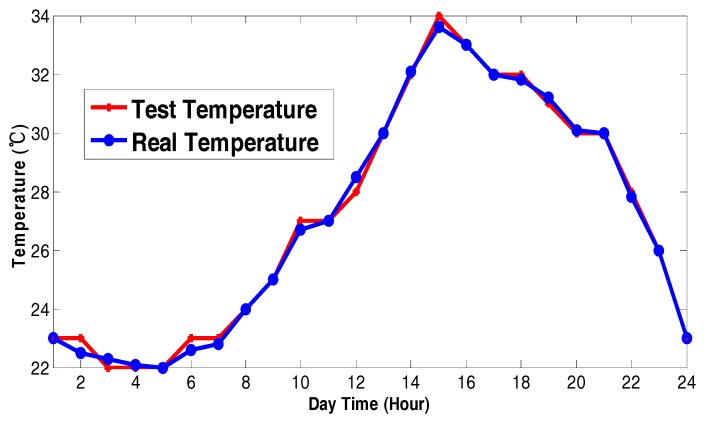
Real-time temperature changes on a sunny day.

**Figure 26:**
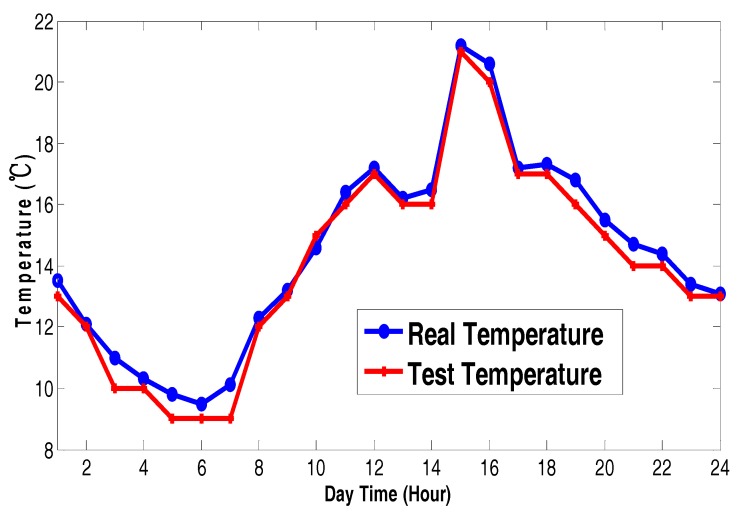
Real-time temperature changes on a cloudy day.

We use a temperature test system based on our solution on an island to collect real temperature changes between sunny and cloudy days. Furthermore, we show the contrast between the test temperature and the actual temperature. Figure 25 and Figure 26 show the temperature change contrast curves of the sunny day and the cloudy day.

The test results show that the sunny day test temperatures are slightly higher than the actual temperature, and the cloudy day test temperatures are slightly lower than the actual temperature due to the less light intensity. The change speed of thermal ink is a little slower on the cloudy day, but its impact on the color sensor sensitivity is negligible. Finally, the test temperature error of both the sunny day and cloudy day is acceptable for the needs of weather reports.

According to the above analysis of the test result, we show that the real-time character of thermal ink fully meets needs of weather report applications. The temperature change of test results is consistent with and the actual temperature change independent of the presence of sunny or cloudy days. The good temperature track performance proves that our solution based on the thermal ink has feasibility and real-time performance. This shows the feasibility of using data fusion to generate accurate real-world measurements.

## 5. Conclusions

To our knowledge, this is the first use of the Agent Based Network Model [14], coming from the business world, to the wireless sensor network area. Like the ABM, we observe network improvement. Whereas Cabral et al. found a significant decrease in reporting time, we found lower total energy consumption and shorter execution time within the network. We discussed our study in two steps. First, we showed that a model based on mobile agent data fusion helps to reduce energy consumption and improve execution time in mobile wireless sensor networks. This result would be irrelevant if data fusion could not produce reliable results. We used data fusion with color sensors to show that, indeed, data fusion can reliably produce measurements even after strong reduction of transmitted data. Together, the advantages of sending fused data and data fusion producing reliable results, show the advantages of our approach over the traditional client/server approach. Since the results of our algorithm are slightly favorable over the LCF routing algorithm with identical payloads, especially with increased network traffic, our algorithm is preferable over LCF routing. In the past, sensor networks used a limited number of stationary sensors and transmission of uncompressed data was efficient enough due to the low overhead of the network. With the continuing increase in number of sensors and the challenges of sensors moving through wireless networks, the cost of increased overhead can be more than offset through proper routing, data fusion, and not collecting more data as soon as the results are good enough. This paper contributes to that quest.

## 6. Future Research

The results of this study will be used in industrial wireless mobile sensor networks for unattended industrial site monitoring, production supervision and management. At the same time, we will also optimize for different network size, network structure, and application objects to improve the reliability and robustness of the system. One of the limitations of our study is the absence of considering the nature of difference in sensor measurements. The non-compatibility phenomenon and improvement method for the multi-protocol agent will be used in heterogeneous environments. We assumed that the fusion protocol itself produces identical results in the measurement model. We will continue to improve the transmission efficiency and rate, and reduce the frequency of the data transmission. A third limitation is the absence of security considerations. Secure computing and secure transmissions need processing time and consume energy. We will work on the security of data fusion of WSN. Finally, we will try to propose standards for the applications and research.

## Figures and Tables

**Table 1:** Selected similarities.

Business Networks	Dam Project	Mobile Wireless Sensor Networks
One client	Dam owner	Sink node
Dependence on all firms	Dependence on suppliers and workers	Dependence on sensor nodes
Need for coordination	Test concrete before use	Fuse data before transmission
Communication problems	Delays in test result availability	Network delays and outages
Disparate ICT systems	Different systems for builders and suppliers	Multiple types of sensors
Disparate data formats between firms	Disparate data formats between builders and suppliers	Different data types for same event
Special middleware systems	Common RFI system	Coordinating nodes (to be discussed)
Project delays	Delayed construction	Delayed detection of events
Project failures	Dam failure/ breach	Failure to detect events

**Table 2:** Application proxy data structure.

**Identification**	**Data Space**	**Service Know-How**	**Itinerary**

**Table 3:** DF information format.

**Node ID**	**Remaining Energy**	**Signal Energy**	**Longitude**	**Latitude**

**Table 4:** Notification packet format.

**Id**	**Source_Id**	**Longitude**	**Latitude**	**Remaining Energy**	**Signal Energy**	**Type_Info**

**Table 5:** Mobile agent data format.

**Id**	**Code**	**Task**	**Result Space**	**Iterary**	**Agent_Type**

**Table 6:** Process of sensor node.

**Process: Processing by Sensor Nodes**
1: Nodes detect that event occurs 2: Nodes = Collect(data) 3: Nodes send Notification Packets 4: RMS = Receive(Packets) 5: do{ 6: RMS makes sure that there is a certain type of events 7: MA = Wait(Mobile_Agents) 8: The data is fused here 9: Mobile_Agents clear old data and store new data. 10: }while(old data isn’t cleared) 11: Event ends 12: Nodes stop collecting data
